# Metaheuristic-enhanced deep learning for monthly pan evaporation prediction under limited climatic data

**DOI:** 10.1038/s41598-026-51071-3

**Published:** 2026-05-02

**Authors:** Ozgur Kisi, Rana Muhammad Adnan, Mohammad Zounemat-Kermani, Saad Sh. Sammen, Adil Masood, Christoph Külls

**Affiliations:** 1 Department of Civil Engineering, Lübeck University of Applied Sciences, 23562 Lübeck, Germany; 2 Department of Civil Engineering, Ilia State University, 0162 Tbilisi, Georgia; 3 School of Civil, Environmental and Architectural Engineering, Korea University, Seoul, 02841 South Korea; 4 Department of Anatomy, Saveetha Medical College and Hospital, Saveetha Institute of Medical and Technical Sciences, Chennai, India; 5 Water Science and Environmental Research Centre, College of Chemistry and Environmental Engineering, Shenzhen University, Shenzhen, People’s Republic of China; 6 Department of Civil Engineering, Shahid Bahonar University of Kerman, Kerman, Iran; 7 Department of Civil Engineering, College of Engineering, Diyala University, Baquba, Diyala Governorate Iraq; 8 TERI School of Advanced Studies, New Delhi, 110070 India

**Keywords:** Pan evaporation, Deep learning, Artificial Protozoa Optimizer, Dung Beetle Optimizer, Metaheuristic algorithms, Limited inputs, Climate sciences, Computational biology and bioinformatics, Environmental sciences, Mathematics and computing

## Abstract

This study introduces two recently developed bio-inspired metaheuristic algorithms, Artificial Protozoa Optimizer (APO, 2024) and Dung Beetle Optimizer (DBO, 2023), into long short-term memory (LSTM) networks for monthly pan evaporation prediction under limited climatic data. Representing the first application of these algorithms to hydrological modeling, these models integrate APO and DBO into the LSTM framework to optimize hyperparameters and enhance accuracy and generalization. Their performance is benchmarked against the standard LSTM and two established hybrids, LSTM-GWO and LSTM-HHO. A case study in southeast China using 40 years of data from two stations shows that both LSTM-APO and LSTM-DBO consistently outperform the alternatives across three data-splitting scenarios (M1, M2, M3). For the best test case (M3, Station 1), LSTM-APO reduced RMSE and MAE by 46.5% and 47.2%, respectively, compared to the best LSTM, while in Station 2 (M2) it achieved reductions of 43.9% and 40.7%, with gains of about 9% in R² and NSE. LSTM-DBO also yielded notable improvements, reducing errors by 20–30% and demonstrating robust predictive stability. Visual analyses confirm that LSTM-APO provides predictions closely aligned with observations, with LSTM-DBO performing comparably well. These findings highlight the role of metaheuristic optimization in boosting LSTM performance for nonlinear evaporation processes with sparse inputs. Overall, APO- and DBO-based hybrids show strong promise for reliable pan evaporation forecasting. Future research should assess their real-time applicability and transferability across diverse climates.

**Supplementary Information:**

The online version contains supplementary material available at 10.1038/s41598-026-51071-3.

## Introduction

Evaporation is an essential part of the hydrological cycle. It represents the change of liquid water into vapor due to pressure differences between the water surface and the atmosphere^1^. This process is quite complex and challenging to predict because it interacts with water surfaces, land, atmospheric processes, and vegetation^2^. Getting a precise estimate requires effective modeling techniques to assist with water management, farming, and environmental protection. There are two basic methods for estimation: direct and indirect^3^.

Pan evaporation (Ep) is a standard direct method. While practical, it suffers from instrument errors and maintenance problems^4^. Indirect methods connect Ep to climate variables through empirical or semi-empirical equations, but their use is often limited by incomplete data^5^. The non-linear and unstable nature of evaporation makes accurate modeling even harder. This has led to a rise in the use of machine learning (ML) and deep learning (DL) approaches. For example, Goyal et al.^6^ reported that LS-SVR and fuzzy logic improved daily Ep estimation. Majhi and Naidu^7^ found that artificial neural networks (ANN) were more accurate than empirical methods. Later, Majhi et al.^8^ demonstrated that deep LSTM models trained on long-term daily data could provide strong and reliable Ep predictions.

Recent studies have emphasized the success of hybrid metaheuristic models in enhancing prediction accuracy. Guan et al.^9^ found that SVR-KHA outperformed standalone SVR in Iran. Keshtegar et al.^10^ proposed SVR-RSM in Algeria, achieving accurate estimates of Ep. Wang et al.^11^ confirmed that SSA-KNEA outperformed traditional models in China. The integration of LSTM with metaheuristic optimization has recently gained significant attention in hydrological prediction: Apak et al. (2025) developed an incremental attention network combining LSTM with Chaos optimization for streamflow prediction, demonstrating substantial improvements over standalone models^12^. Similarly, Sarıgöl et al. (2024) applied LSTM with advanced optimization techniques for streamflow forecasting, reporting enhanced predictive accuracy. These studies establish that metaheuristic optimization is essential for maximizing LSTM performance in water resources applications^13^.

Other advances include LSTM-GWO in Turkey, which surpassed both individual ML and empirical models, and NCA-LSTM in Australia, which produced more accurate Ep forecasts during drought conditions.

Hybrid metaheuristic models have demonstrated high accuracy in forecasting pan evaporation. They achieve low RMSE and high R² values. Woo et al.^14^ assessed an LSTM-based hybrid model for simulating evapotranspiration using several spatial climatological datasets. The results showed that daily performance was highest in snow-influenced continental climates; however, in arid and high-latitude regions, larger discrepancies were reported. Similarly, Jayasinghe et al.^15^ created a hybrid LSTM (NCA-LSTM) for drought-affected areas of Australia. This model outperformed single ML methods in simulating Ep.

Recent developments further emphasize the promise and higher potential of hybrid models in modeling hydrological phenomena^16^. In the field of evaporation modeling, Zerouali et al.^17^ developed an LSTM hybrid model with the Binary Al-Biruni Earth Radius for Epan estimation across diverse climatic zones in Algeria, resulting in up to a 97.54% improvement in RMSE compared to the traditional LSTM on daily time scales. More recently, Alsumaiei applied several hybrid DL methods, including LSTM and stepwise linear regression, to predict evaporation in arid climates and demonstrated that hybrid ML models outperformed their individual counterparts^18^. Al-Juboori^19^ proposed a CCNN-GLM hybrid model for daily pan evaporation in semi-arid regions of Iraq, yielding R² values of 0.95 and 0.93 for training and testing, respectively. Farzad et al.^20^ considered individual and hybrid LSTM models (such as LSTM-BH, LSTM-MPA, and LSTM-MVO) for monthly reservoir evaporation in Iran and reported that the LSTM model integrated with the Marine Predator algorithm achieved higher accuracy. Consistent with the general findings of these studies, other recent works have also reported the superior accuracy of hybrid ML methods for modeling and predicting evaporation across different climatic zones^21–24^.

Overall, the review of the literature indicates that these novel pan evaporation prediction techniques can better model the evaporation process across various locations and achieve superior results compared to traditional standalone models. The superiority of these advanced hybrid techniques stems from their enhanced generalization, improved accuracy, and lower computational complexity compared to individual models.

Recent research has illuminated the potential uses of new bio-inspired algorithms, such as APO^25^ and DBO^26^, to enhance the optimization capabilities of the deep learning model LSTM. The APO provides a strong optimization approach that has been inspired by a protozoa’s behaviors, particularly their foraging, dormancy, and reproducing, and is capable of balancing exploration and exploitation for enhanced solution accuracy. The DBO has been inspired by the behaviors exhibited by the dung beetle when it rolls balls, dances, forages, steals, and reproduces, and has been shown to perform competitively on global optimization and engineering design problems. Although several studies have employed ML models to estimate pan evaporation across different regions of the globe, the combined application of advanced metaheuristic techniques such as APO and DBO, along with deep learning for pan evaporation prediction, remains largely unexplored. This study begins to investigate and assess two novel hybrid DL models, LSTM-APO and LSTM-DBO, compared to single LSTM, LSTM-HHO, and LSTM-GWO for forecasting monthly pan evaporation from sparse climatic data.

Despite advances in hybrid LSTM models, three critical challenges and research gaps remain inadequately addressed in the literature: (1) Data Availability: Limited input to LSTMs has not been well characterized, and many water management activities occur where the LSTM has limited input; (2) Mechanistic Justification: LSTM hybrids are often trained to established optimization algorithms (GWO/HHO), without consideration of the characteristics of the problem at hand, while recent advancements in optimization (APO/DBO), with very different search strategies, have not been applied in any hydrologic application; (3) Robustness: Single Train-Test evaluations do not capture the sensitivity of optimizers to data availability or provide an indication of an optimizer’s ability to reliably perform across operational settings (i.e., 70–80% training/validation). Based on the literature review and the above-mentioned research gaps, this study’s two primary contributions are: (i) domain novelty: introducing APO and DBO for the first time into hydrological modeling via pan evaporation prediction while broadening their usage beyond engineering and energy); and (ii) methodological insight: illustrating mechanistic advantages of dormancy-driven adaptive search with APO and multi-role behavioral specialization with DBO over GWO’s hierarchical framework and HHO’s cooperative-hunting framework for limited input time series forecasting).

A case study in southeast China, using 40 years of historical meteorological data from two stations, evaluates these models with three data-splitting schemes (M1, M2, M3). Building on these observations, the present study seeks to achieve the following objectives:

(i) To create a novel hybrid DL method called LSTM-APO and LSTM-DBO for estimating Epan using limited data.
(ii) To evaluate the forecasting abilities of different individual and hybrid LSTM-APO, LSTM-DBO, LSTM-HHO, and LSTM-GWO models for Epan estimation.
(iii) To evaluate the transferability of the newly developed and proposed LSTM-APO and LSTM-DBO models to diverse climatic regions.

Since evaporation, a vital component of the hydrologic cycle, has significant consequences for water resource management, agriculture, and ecosystem sustainability, accurately forecasting Epan is crucial for both research and applied endeavors. While there has been progress in the use of ML and hybrid optimization models for pan evaporation, many avenues remain unexplored to maximize the advantages of newly developed metaheuristic algorithms in evaporation modeling. Hence, this study advances the understanding of pan evaporation modeling by introducing conventional (LSTM-HHO & LSTM-GWO) and novel (LSTM-APO & LSTM-DBO) integrated deep learning models. It helps fill the gap in knowledge about improving prediction accuracy and robustness in environments where data are limited, while also providing new methodological pathways for the water sciences. The outcomes of the research are expected to promote better decision-making across diverse climatic settings, thereby contributing fundamentally to the advancement of hydrological modeling and the sustainable management of global water resources.

## Case study

**Fig. 1:**
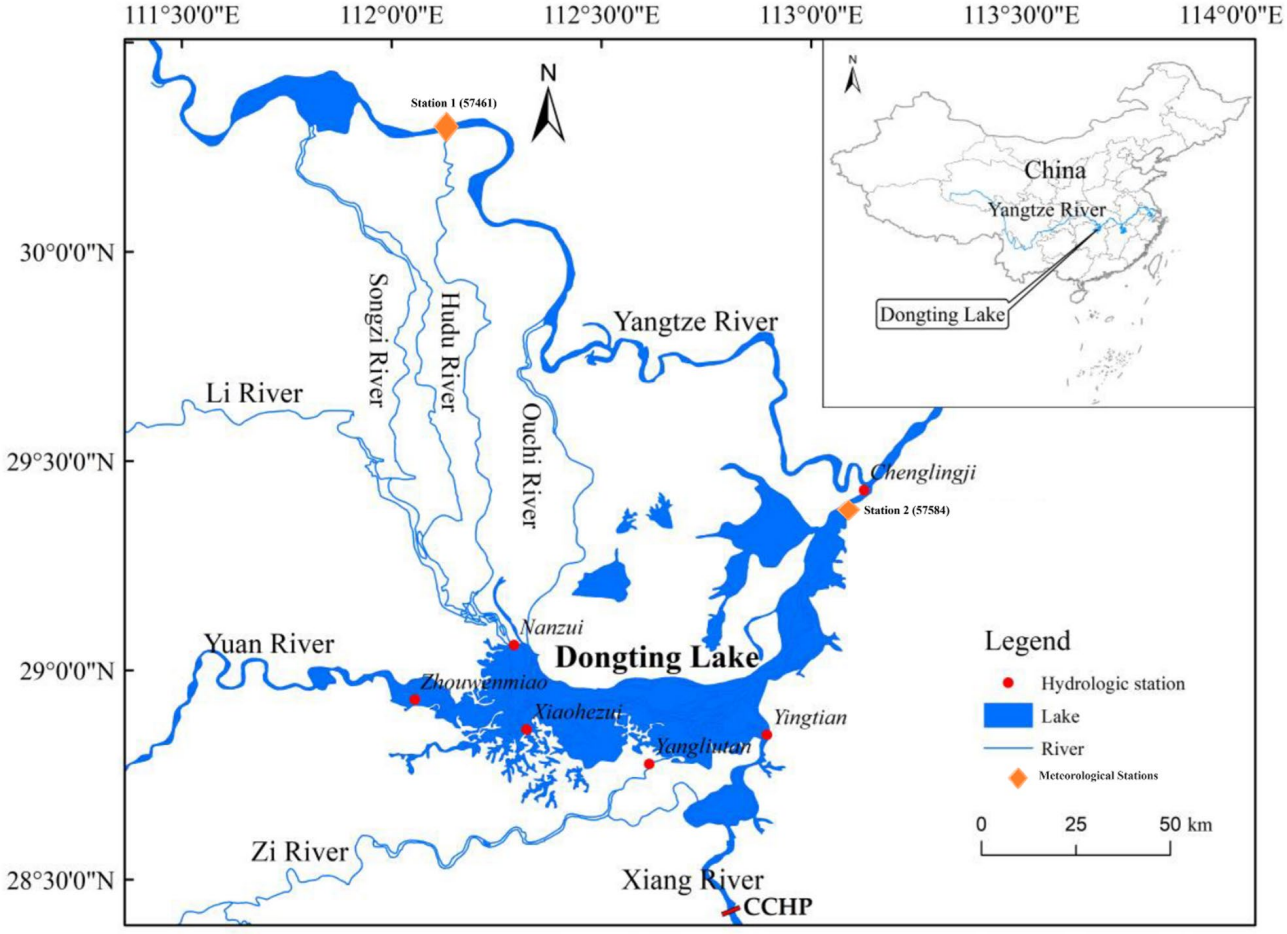
The location of stations in the study area. Map created by the authors using ArcGIS Desktop 10.8.1 (Esri, 2020; https://www.esri.com/en-us/arcgis/products/arcgis-desktop/overview).

In this study, southeast region of China is chosen as shown in Fig. 1. This region is situated between 20 and 32 N and 108–123 E with the landforms in the form of mountain and low hills. This region is selected due to its key role in agricultural products, especially for Rice. In addition to this, the world’s second-largest freshwater lake, Dongting Lake, is also situated in this region. This region is also selected because it represents a complex river–lake interaction system, where the Yangtze River connects with Dongting Lake through multiple inlets and outlets, forming a typical passing-lake basin^27,28^. The climate is subtropical monsoon with climate with hot, humid summer whereas warm and moist winter with an annual precipitation ranging from 1340 mm to 1545 mm concentrated from April to June, leading to pronounced seasonal hydrological variability and frequent overlapping floods from the Yangtze and local tributaries^29,30^. In recent decades, the region has faced increasing water‐related hazards, including floods, waterlogging, and seasonal droughts, accompanied by rising socioeconomic losses^31,32^.

This region also comprises of two key provinces of China i.e. Hubei and Hunan. Both basins make important contributions to China’s economic development. However, due to uneven distribution of precipitation in the region, both provinces face frequently flood and drought events. For the present study, two meteorologicalstations were selected for analysis as illustrated in Fig. 1 (Station 1 = Station 57461 and Station 2 = Station 57584). Due to key importance of both provinces, station 1 (station 57461) is selected from Hubei province whereas station 2 (station 57584) was selected from Hunan Province. Monthly observations of pan evaporation, maximum and minimum air temperature covering a 40-year period were obtained from the China Meteorological Administration (CMA). The data was divided into three different data splitting scenarios to better analyze the effect of data on prediction accuracy. The three splitting strategies adopted in this study for better evaluation of methods are; M1 (70 training and 30% testing), M2 (75 training and 25% testing), and M3 (80 training and 20% testing). Table 1 reports the brief statistical of the data of both climatic stations. The parameters reported in the table are T_min_ (°C): minimum temperature; T_max_ (°C): maximum temperature; R_a_ (MJ/m^2^): radiation; and E_pan_ (mm): pan evaporation. Extraterrestrial radiation (Ra) was calculated using standard FAO-56 formulas that take into account latitude, the day of the year, and solar geometry. It represents the solar radiation that reaches the top of the atmosphere and does not consider local atmospheric conditions, such as cloud cover, humidity, or aerosols.

**Table 1:** Brief statistics of the data of two climatic stations.

	Station 1				Station 2				
	Tmin (°C)	Tmax (°C)	Ra (MJ/m^2^)	Epan (mm)	Tmin (°C)	Tmax (°C)		Ra (MJ/m^2^)	Epan (mm)
Mean	13.32	21.38	31.40	3.62	14.36	20.75		31.89	3.94
Min.	−2.36	4.45	19.75	0.884	−0.935	2.85		20.80	0.911
Max	26.04	36.28	41.13	10.62	27.95	35.17		40.93	11.12
Skewness	−0.102	−0.135	−0.185	0.616	−0.05	−0.12		−0.21	0.831
Std. dev.	7.94	8.45	7.65	1.81	8.38	8.51		7.21	2.17

## Methodology

**Fig. 2:**
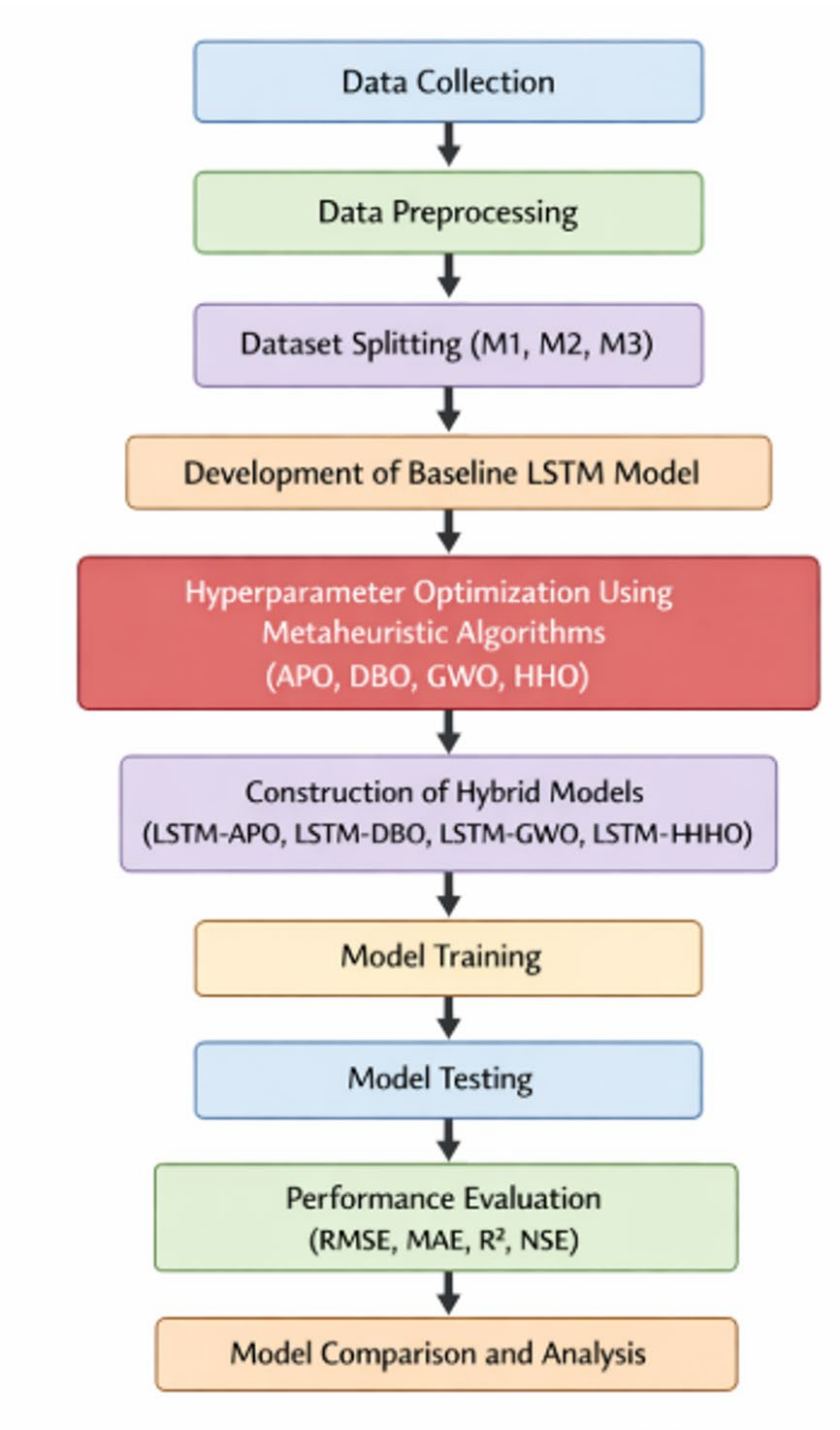
Workflow of the proposed methodology.

To clarify the proposed methodology, the study’s overall workflow is shown in Fig. 2. The process starts with data collection and preprocessing. Next, the dataset is divided into three scenarios: M1, M2, and M3. A baseline LSTM model is developed first. Then, its hyperparameters are optimized using various metaheuristic algorithms: APO, DBO, GWO, and HHO. Using these optimized parameters, hybrid models are created and trained. Finally, the models’ performance is assessed using statistical metrics, including RMSE, MAE, R², and NSE. A thorough comparison is carried out.

### Data preprocessing

Before model development, the dataset went through several preprocessing steps to ensure quality and consistency. First, the raw meteorological variables, including minimum temperature (Tmin), maximum temperature (Tmax), extraterrestrial radiation (Ra), and pan evaporation (Epan), were checked for completeness and consistency. Any irregular or inconsistent records were addressed to prevent bias during model training. To improve numerical stability and the learning efficiency of the LSTM models, all input variables were normalized to a common scale. This step is important in deep learning because it stops larger variables from dominating the training process.

Additionally, various input combinations were created using different sets of climatic variables, such as Tmin, Tmax, and Ra, to study their effect on model performance. These combinations aimed to evaluate how well the models perform with limited data. Finally, the preprocessed dataset was divided into three data-splitting scenarios (M1, M2, and M3) to test the model’s performance under different training and testing conditions.

### Selection of input variables

The choice of input variables depended on data availability and their relevance to evaporation processes. Minimum temperature (Tmin) and maximum temperature (Tmax) were chosen as main inputs because they directly affect vapor pressure deficit and thermal conditions, which drive evaporation. Extraterrestrial radiation (Ra) was added as another variable to represent the theoretical solar energy incident at the top of the atmosphere.

To evaluate the effect of input structure with limited data, we created several input scenarios using different combinations of these variables, including Tmin, Tmax; Tmin and Ra; Tmax and Ra; and Tmin, Tmax, and Ra. This method helps assess model performance and the contribution of each variable to evaporation prediction. This approach is vital in data-scarce environments, where identifying the most informative, non-redundant variables is crucial for building strong, dependable, and adaptable models.

### Long short term memory

The LSTM represents an advanced variant of RNN, widely used for modeling sequential and temporal data. LSTMs were introduced for the first time in 1997 by Hochreiter and Schmidhuber (1997) as a remedy for the vanishing gradient problem^33^. However, LSTM performance is highly sensitive to hyperparameter selection, including learning rates, hidden unit counts, and dropout rates. Recent studies have demonstrated that systematic hyperparameter optimization can significantly enhance LSTM predictive capabilities across diverse applications^34^. It has become a powerful tool and used by researchers in various applications for time series prediction where it has the capability preserving important information over extended time spans^35–39^. LSTM introduced a novel cell state, which serves as a memory unit to capture and store relevant information over long time intervals. This cell state is regulated by three gating mechanisms: the input, forget, and output gates.

The forgetting gate is regulating the degree to which components of the cell state vector (C_t−1_) have to be eliminated.

1 $$\:{f}_{t}=\:\sigma\:({W}_{fxt}+{U}_{f{h}_{t-1}}+{b}_{f})$$

Where *f*_t_ is the resultant vector and its value lie inside the interval (0,1), σ is the Sigmoid function, *W*_*f*_, *U*_*f*_ are the two modifiable matrices of wight and *b*_*f*_ is the bias factor. After that, in the input gate and by using the present value of (*x*_*t*_) and the previous hidden state (*h*_*t−1*_) which is provided by the following Eq. (2), a possible updating vector for the cell state is computed.

2 $$\:\stackrel{\sim}{{c}_{t}}=\mathrm{t}\mathrm{a}\mathrm{n}\mathrm{h}({W}_{\stackrel{\sim}{c}{x}_{t}}+{U}_{\stackrel{\sim}{c}{h}_{t-1}}+{b}_{\stackrel{\sim}{c}})$$

*Where* $\:\stackrel{\sim}{{c}_{t}}$ is a vector in the interval *(0*,*1)*, $\:{W}_{\stackrel{\sim}{c}{x}_{t}},{U}_{\stackrel{\sim}{c}{h}_{t-1}}and\:{b}_{\stackrel{\sim}{c}}$ are another group values of wight matrices and bias. Additionally, In this stage, the input gate is compute using the following equation 

3 $$\:{i}_{t}=\:\sigma\:({W}_{i{x}_{t}}+{U}_{i{h}_{t-1}}+{b}_{i})$$

Where *i*_*t*_ is the vactor in the interval (0,1), *W*_*i*_, *U*_*i*_ and *b*_*i*_ are set value of the wight matrices and bias. According to the results of the above three equation., the value of the cell state is modified following Eq. (4)

4 $$C_{t} = \:f_{t} \odot C_{{t - 1}} + i_{t} \odot \mathop {c_{t} }\limits^{\sim }$$

Where (ʘ) denote the element wise mutiplication.

According to Eq. 5, the information in *C*_*t−1*_ is either forgotten (f_t_ = 0) or mentioned (f_t_ =1). The samething will happen with the *C*_*t*_, where the information will forgoteen when the value of *i*_*t*_ is 0 and it will kept when the value of *i*_*t*_ is one.

After that, the output gate is rgulate the data flow from the cell state to the new hidden state using the following equation:

5 $$\:{O}_{t}=\:\sigma\:\left({W}_{o{x}_{t}}+{U}_{o{h}_{t-1}}+{b}_{o}\right)$$

Where *W*_*o*_, *U*_*o*_ *and b*_*o*_ are the set values of wight matrices and bias.

The new hidden state is computed using the Eq. (4) *and* Eq. (5) *as folloing*:

6 $$h_{t} = {\mathrm{tanh}}\left( {C_{t} } \right) \odot O_{t}$$

**Fig. 3:**
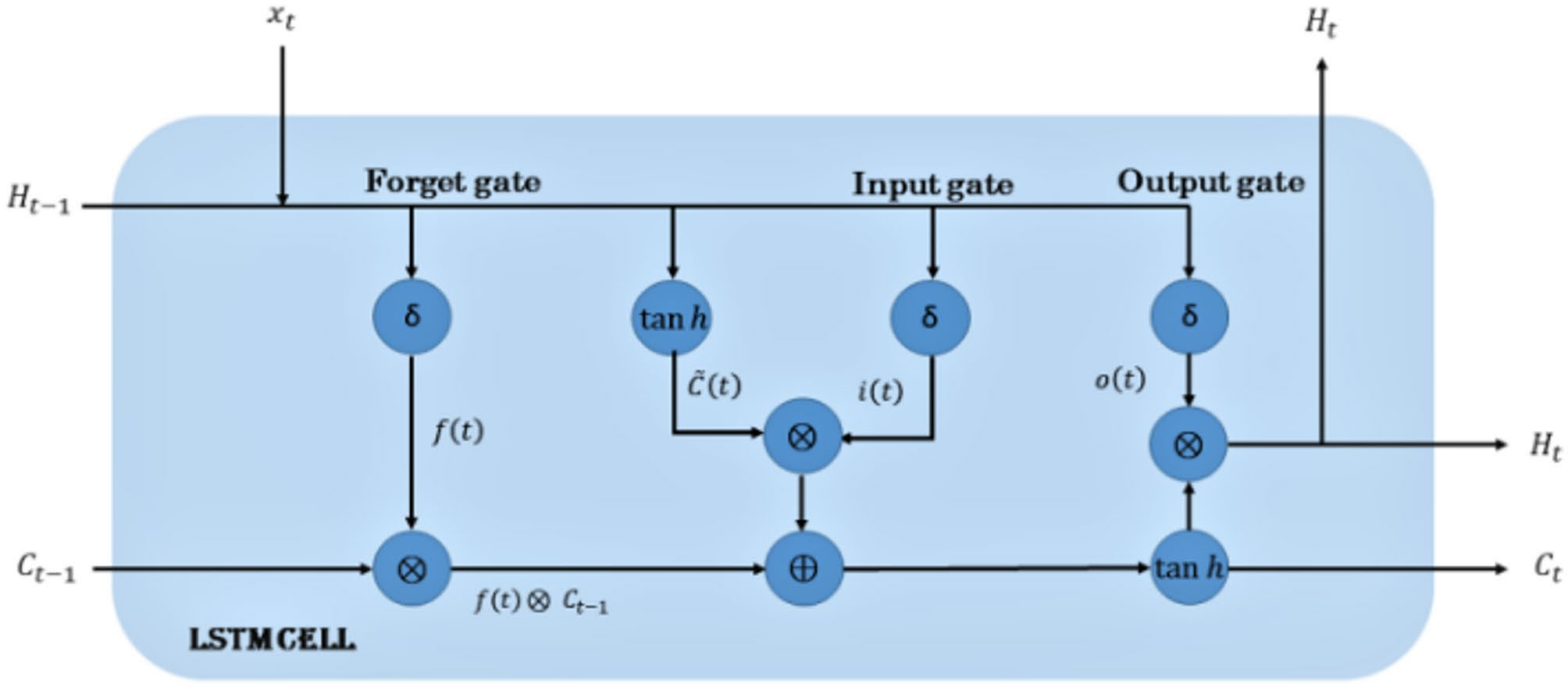
The architecture of LSTM.

Figure 3 illustrates the architecture of the network. These gating mechanisms enable LSTMs to selectively remember and forget information, allowing them capturing both short- and long-term dependencies within the data. LSTMs have several advantages over traditional RNN architectures. Firstly, they can handle long sequences without suffering from the vanishing gradient problem. This makes them particularly effective for tasks that involve long-term dependencies. Secondly, LSTMs have a capability of learning, recognizing and remembering important patterns in the data, which makes them useful for tasks that needs understanding and reasoning. The interaction between the gating components and the memory cell enables the network to dynamically regulate information flow—retaining relevant inputs while discarding less useful ones—thereby ensuring strong adaptability across diverse sequence modeling applications.

### Optimization algorithms

One of the challenges in training LSTM models is finding the optimal set of hyperparameters that can lead to better performance. This is where optimization algorithms come into play. In this study, several optimization algorithms are used to enhance the LSTM performance such as Harris Hawks Optimization (HHO), Grey Wolf Optimization (GWO) and Sea Horse Optimization (SHO).

#### Harris Hawks Optimization (HHO)

The HHO algorithm introduced by Heidari et al. in 2019 is a nature-inspired metaheuristic derived from the cooperative hunting strategies of Harris’s hawks, a species of raptors natives to the southwestern US. This algorithm emulates the collective and dynamic hunting tactics of hawks to explore and exploit the search space efficiently in solving complex optimization problems. By leveraging the collective intelligence of the flock, the algorithm aims to find the best solution for the optimization problem.

**Fig. 4:**
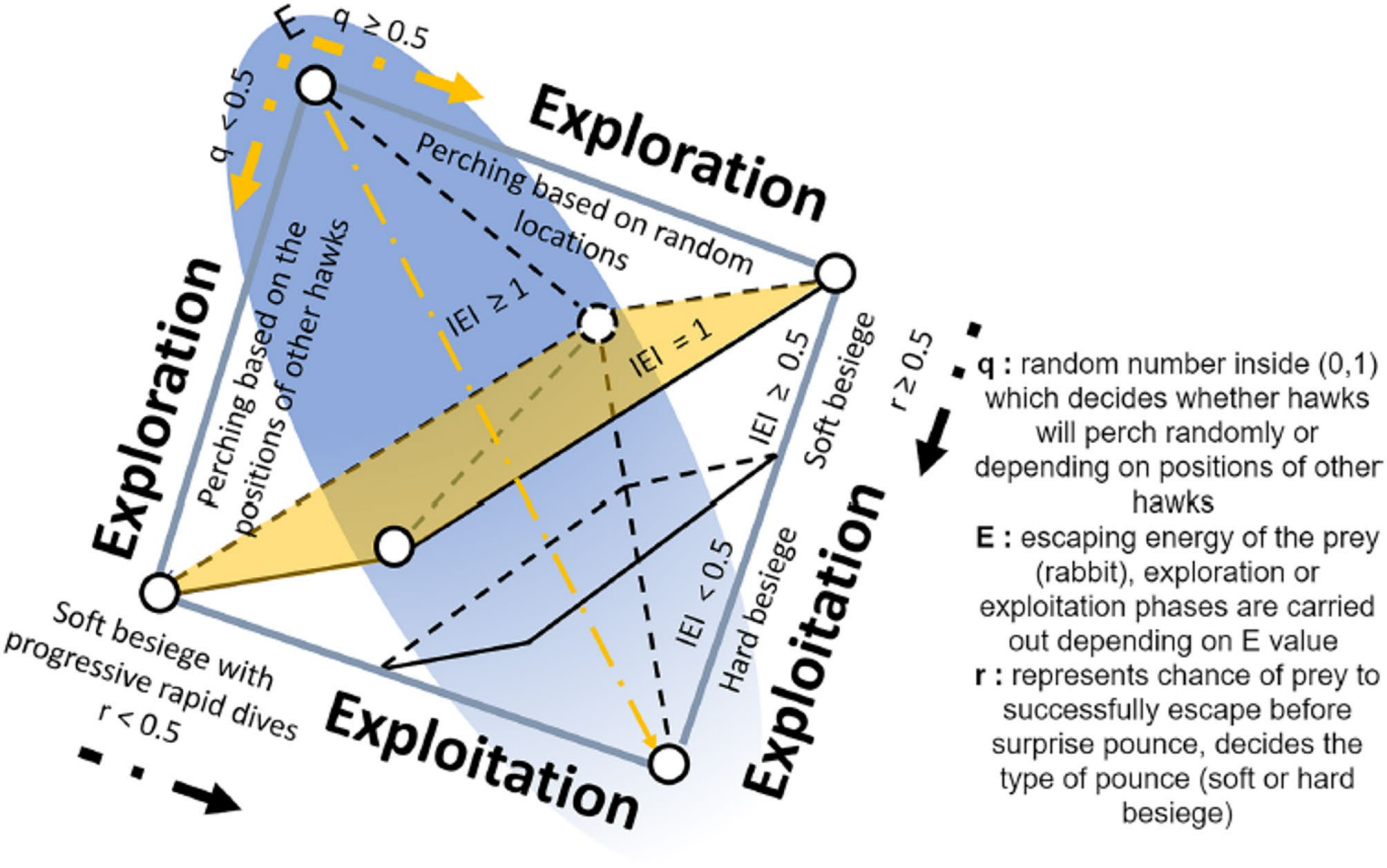
The exploration and exploitation of the HHO algorithm^7^.

Similar to other population-based methods such as PSO GWO, HHO relies on the collaborative intelligence of multiple search agents to locate near-optimal solutions. This algorithm has been successfully employed in diverse areas, including engineering design^41^, classification^42^ and ML optimization^43,44^. Owing to its hierarchical design and adaptive switching between exploration and exploitation, HHO has demonstrated superior convergence speed and robustness compared to several conventional algorithms. Its conceptual workflow is illustrated in Fig. 4, and further algorithmic details can be found in^40^.

#### Grey Wolf Optimization

The GWO, introduced by Mirjalili et al. (2014)^45^, is another bio-inspired optimization approach modeled after the leadership hierarchy and cooperative hunting behavior of grey wolves. GWO’s appeal lies in its conceptual simplicity, few control parameters, and high search efficiency, which have led to successful applications in image processing^46,47^ and forecasting tasks^48,49^.

**Fig. 5:**
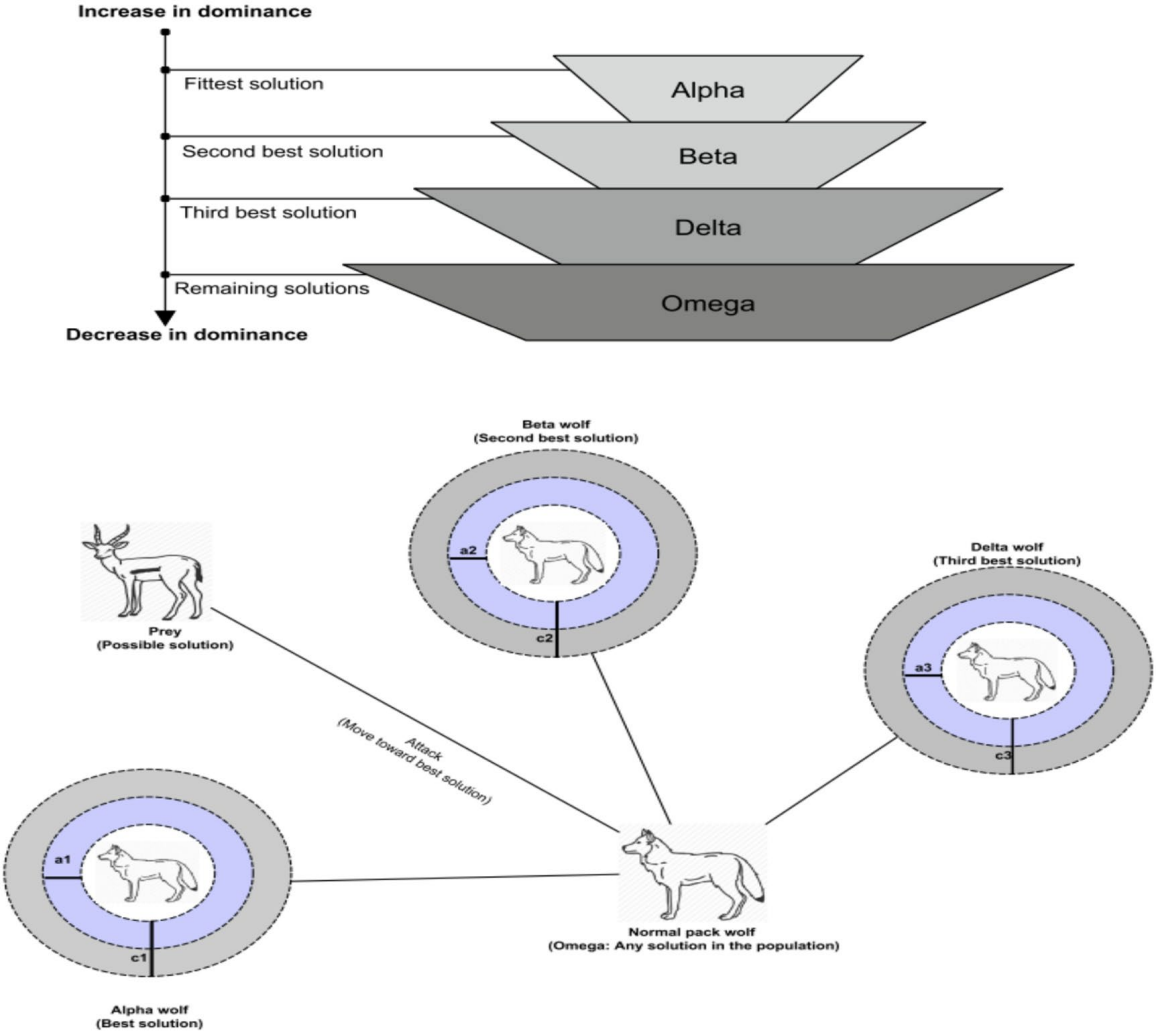
**a** Hierarchy of grey wolves, **b** location update of ω wolves according to other wolves (α, β and δ)^17^.

In this algorithm, each candidate solution—referred to as a wolf—represents a potential position in the search landscape. The hierarchy consists of four ranks: alpha (α), beta (β), delta (δ) and omega (ω) wolves as shown in Fig. 5a and b. These wolves collaborate in a hierarchical manner to hunt their prey effectively. The main stages of the algorithm are summarized as follows:

- Initialization: Initialize the positions and fitness of the wolves randomly within the search space.
- Prey search: Each wolf searches for prey by updating its position based on its current position and the positions of the other wolves. The updated position is determined by the formula:

7 $$\:{X}_{(t+1)}=\:{X}_{P\left(t\right)}-A*D$$

Where: *t* where t refers the existing iteration, *X*_*(t+1)*_ is the new position of wolf, *X*_*P(t)*_ is the position vector of prey, *A* is a random vector, and *D* is the distance between the current wolf and the prey. *A* and *D* and could be calculated as following:

8 $$\:A=2a*{r}_{1}-a$$

9 $$\:D=\:\left|C*{X}_{Pt}-\:{X}_{t}\right|$$

10 $$\:C=2*{r}_{2}$$

Where *a* ranged in (0–2) and *r*_*1*_ and *r*_*2*_ ranged in (0–1).

- Update the hierarchy: After the prey search, the hierarchy is updated based on the fitness values of the wolves. The alpha, beta, and delta wolves are updated based on their fitness values, while the omega wolf is updated according to the other three wolves.
- Boundary handling: If a wolf moves outside the search space, its position is adjusted to the boundary.
- Fitness evaluation: Calculate the fitness values of the wolves based on the objective function of the optimization problem.
- Termination: The process continues iteratively through Steps 2–5 until a predefined stopping condition is fulfilled, such as achieving the maximum iteration count or attaining a satisfactory fitness value.

#### Artificial Protozoa Optimizer (APO)

The APO is a new bio-inspired metaheuristic that was introduced by Wang and collaborators in 2024^50^. The algorithm is inspired by the euglena protozoa’s survival strategies and simulates key behaviors such as (i) foraging, (ii) dormancy, and (iii) reproduction during optimization to maintain an effective balance between exploration and exploitation during the optimization process. In general, Protozoa, as simple unicellular organisms, have an autotrophic (photosynthesis-based) and heterotrophic (absorption-based) foraging, dormancy during stress, and asexual reproduction through binary fission^50,51^. The APO is used in the context of hyperparameter optimization (APO) to refine deep learning model hyperparameters (here the LSTM) such as learning rates, number of hidden units, and dropout rates to better capture complex nonlinear dependencies. The key to the APO lies in its mathematical abstraction of protozoan movement. Foraging is divided into autotrophic and heterotrophic modes, with position update of the *i*^*th*^ protozoan in autotrophic mode given by:

11 $$X_{i}^{{new}}={X_i}+f \cdot ({X_j} - {X_i}+\frac{1}{{np}}\mathop \sum \limits_{{k=1}}^{{np}} {w_a} \cdot ({X_{k - }} - {X_{k+}})) \odot {M_f}$$

where *f* is the foraging factor, *np* is the number of neighbor pairs, *w*_*a*_ is the autotrophic weight, and *M*_*f*_ is a mapping vector. Heterotrophic foraging searches and follows nutrient-rich areas by updating track to closest nutrient-rich areas. While autotrophic foraging updates based on nutrient rich areas, dormancy resets the stressed protozoa with new random solutions to optimize exploration which promotes more exploration. Reproduction simulates binary fission with controlled perturbations which promote diversity while also ensure diversity and convergence. So far, APO has shown competitive capabilities in benchmark tests, including CEC2022, and in real-world uses where they achieved greater accuracy and stability in solutions compared to GWO and HHO^25^. Through adaptively balancing global search, achieved through dormancy and autotrophic foraging, and local refinement through heterotrophic foraging and reproduction, APO optimally leverage ML models’ hyperparameters even with small datasets.

#### Dung Beetle Optimizer (DBO)

The DBO was first introduced by Xue and Shen in 2023^26^. This algorithm takes inspiration from the broad range of activities done by dung beetles including five satges of: (i) ball-rolling, (ii) dancing, (iii) foraging, (iv) stealing, and (v) reproduction. By its nature, the DBO takes the advantages of both exploration and exploitations procedures. In this sense, the DBO mimics dung beetles’ ecological decomposer roles by balancing global exploration (like rolling and dancing) and local foraging (exploitation, like stealing). The DBO is used to tune hyperparameters, e.g., to set the learning rate and the number of hidden layers in LSTMs, for monthly pan evaporation forecasting improving the model’s ability to learn the non-linear relationships of hydrological data and the temperature and solar radiation inputs^52,53^. The DBO separates the population of beetles by roles in different stages: rolling beetles for navigation, brooders for reproduction, small beetle foragers, and competing thieves. These roles enable DBO to enhance the rate of convergence and the accuracy of the resultant solution. The mathematical framework of DBO implements dung beetle behaviors by iteratively updating positions. In the case of ball-rolling, which imitates navigation using celestial cues, the position of the *i*^*th*^ beetle is updated as:

12 $${x_i}(t+1)={x_i}(t)+\alpha \times k \times {x_i}(t - 1)+b \times {{\boldsymbol{\Delta}}}x$$

where ${{\boldsymbol{\Delta}}}x=\mid {x_i}(t) - {X^w}\mid$ simulates light intensity changes, α is a deflection coefficient $\left( { \pm 1} \right),\,k \in (0,0.2]$is the deflection constant, $b \in (0,1)$is a constant, and *X*^*w*^ is the global worst position. Once the dung beetle has properly discovered a new direction, it should continue rolling the ball in the opposite direction. Therefore, the position of the ball-rolling dung beetle is updated and now reads as follows:

13 $${x_i}(t+1)={x_i}(t)+\tan (\theta ) \times \mid {x_i}(t) - {x_i}(t - 1)\mid , \theta \in [0,\pi ]$$

Reproduction involves defining spawning areas around local best positions X^*^, foraging optimizes around global best X^b^, and stealing updates thieves’ positions to compete for resources. By doing this procedure, the inspired algorithm keeps adapting during the optimization process. In the final optimization stages of DBO, after updating all positions and evaluating fitness values, the algorithm renews the global best solution X^b^ and checks termination criteria.

### Deep learning integration with optimization algorithms

#### LSTM-GWO

The combination of LSTM and GWO, known as LSTM-GWO, has gained attention in recent years for its ability to enhance the performance of LSTM models. By incorporating the GWO algorithm into the training process of LSTM, it is possible to improve the convergence speed and the quality of the learned representations.

The core concept of the LSTM-GWO is to employ the GWO for tuning the weights and biases of the LSTM model during the training phase^54^. The optimization process begins with an initial population of grey wolves, each representing a potential candidate solution. Through iterative position updates inspired by the wolves’ cooperative hunting mechanism, the algorithm explores the search domain to identify improved parameter configurations.

In this hybrid framework, a fitness function is formulated according to the validation performance of the LSTM model. The wolves adjust their locations within the search space based on their fitness scores, progressively converging toward the optimal set of weights and biases that minimize the prediction error between simulated and observed values.

**Fig. 6:**
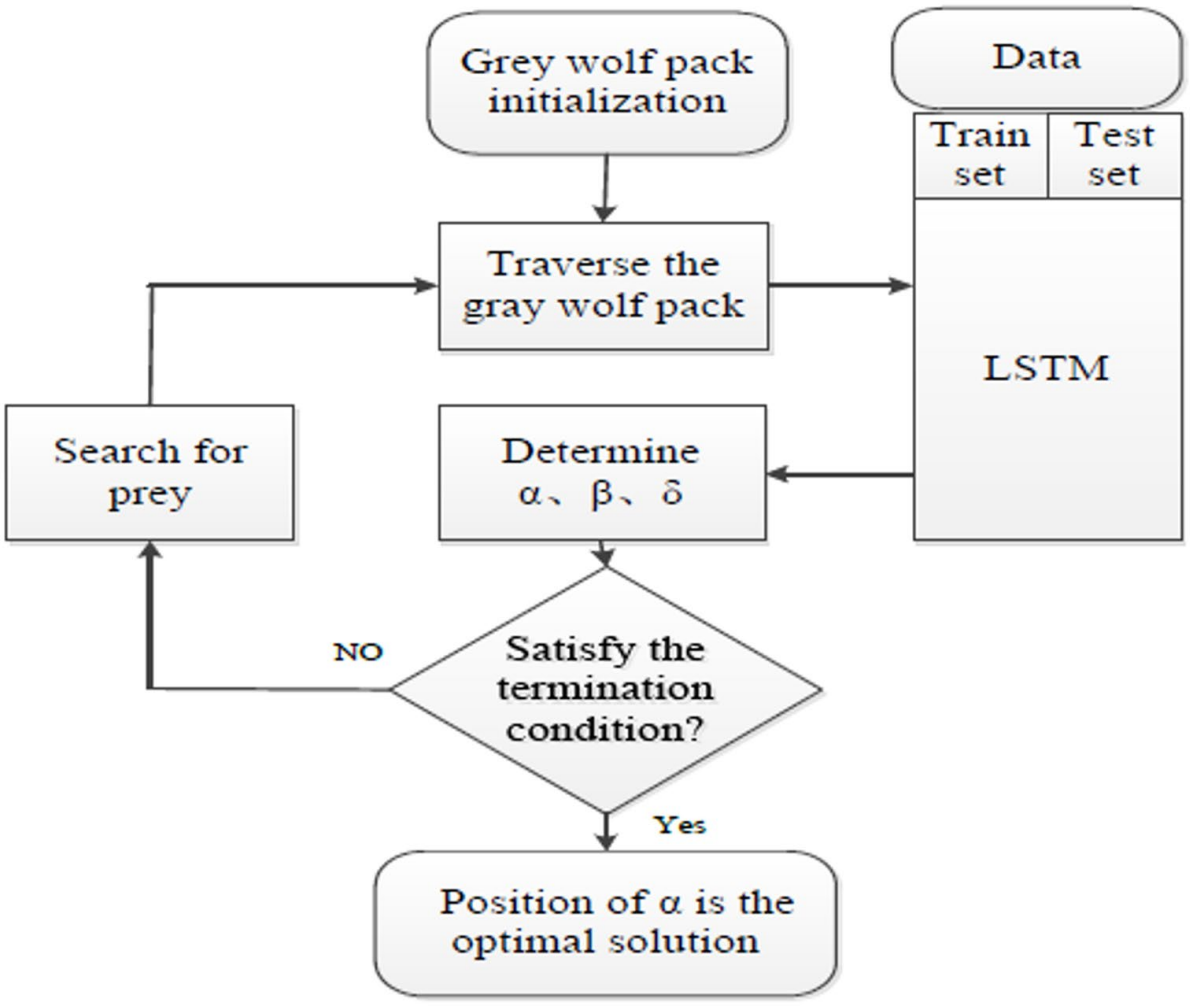
The flowchart of LSTM—GWO^21^.

The advantages of using LSTM-GWO include improved convergence speed, enhanced generalization ability, and effective handling of complex, high-dimensional datasets. T By integrating the GWO algorithm, the model mitigates common drawbacks of conventional gradient-based optimizers, particularly issues related to local minima entrapment and sensitivity to initialization. The overall workflow of the hybrid architecture is illustrated in Fig. 6. Computational workflow of the hybrid LSTM-GWO model is given in the supplementary materials (Table S1).

#### LSTM-HHO

LSTM-HHO, an abbreviation for Long Short Term Memory with Harris Hawks Optimizer, is a hybrid deep learning model that combines the power of LSTM and the optimization technique of Harris Hawks^55^. This unique combination aims to enhance the performance of LSTM models by leveraging the benefits of the Harris Hawks optimization algorithm.

The main idea behind LSTM-HHO is to improve the training efficiency and convergence speed of LSTM models by employing the Harris Hawks optimization algorithm. This hybrid model not only benefits from the powerful memory retention capabilities of LSTM but also takes advantage of the optimization capabilities of the Harris Hawks optimizer.

**Fig. 7:**
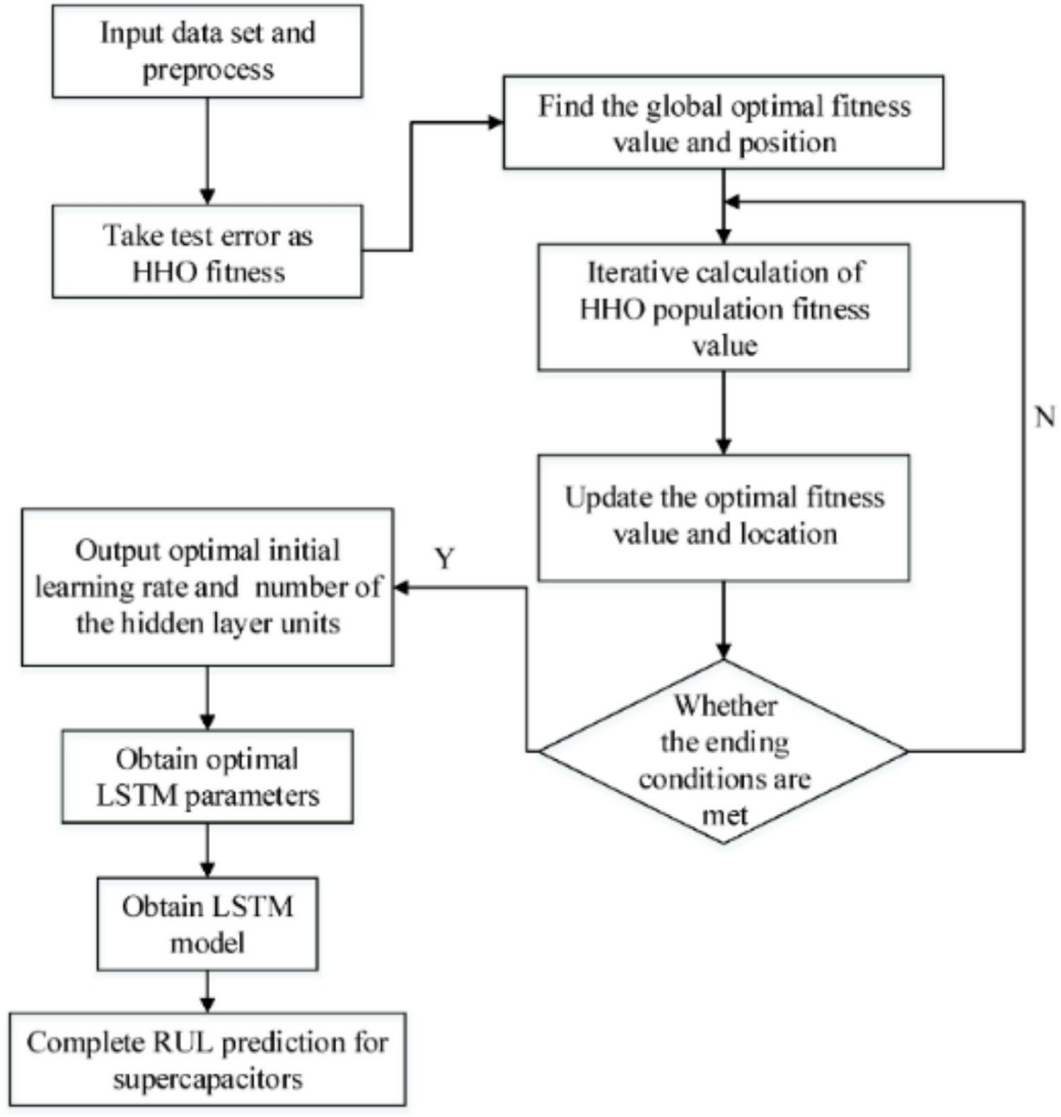
The flowchart of LSTM—HHO model^22^.

The training process of LSTM-HHO involves two main steps: the forward pass and the backward pass. During the forward pass, the input data is fed into the LSTM layers, and the model makes predictions based on the current weights and biases. The backward pass utilizes the Harris Hawks optimization algorithm to update the weights and biases of the LSTM model, aiming to minimize the loss function. Figure 7 shows the flowchart of hybrid model (LSTM-HHO). Computational workflow of the hybrid LSTM-HHO model is given in the supplementary materials (Table S2).

#### LSTM-APO

The integration of LSTM and APO (so called LSTM-APO developed in this research) represents a novel hybrid deep learning model. Similar to other applied integrated LSTM models, it was decided to cope with the potential shortcomings of the individual LSTM model (such as sensitivity to hyperparameter selection and potential overfitting in nonlinear hydrological processes) by integrating the bio-inspired APO algorithm into the LSTM architecture. As previously described, the APO, inspired by protozoan behaviors like foraging, dormancy, and reproduction, provides a robust mechanism for global and local search. This can lead to efficient tuning of LSTM hyperparameters including learning rate, number of hidden units, batch size, and dropout rate. This hybridization leverages APO’s ability to balance exploration (through autotrophic foraging and dormancy) and exploitation (via heterotrophic foraging and reproduction), resulting in improved model generalization and stability.

**Fig. 8:**
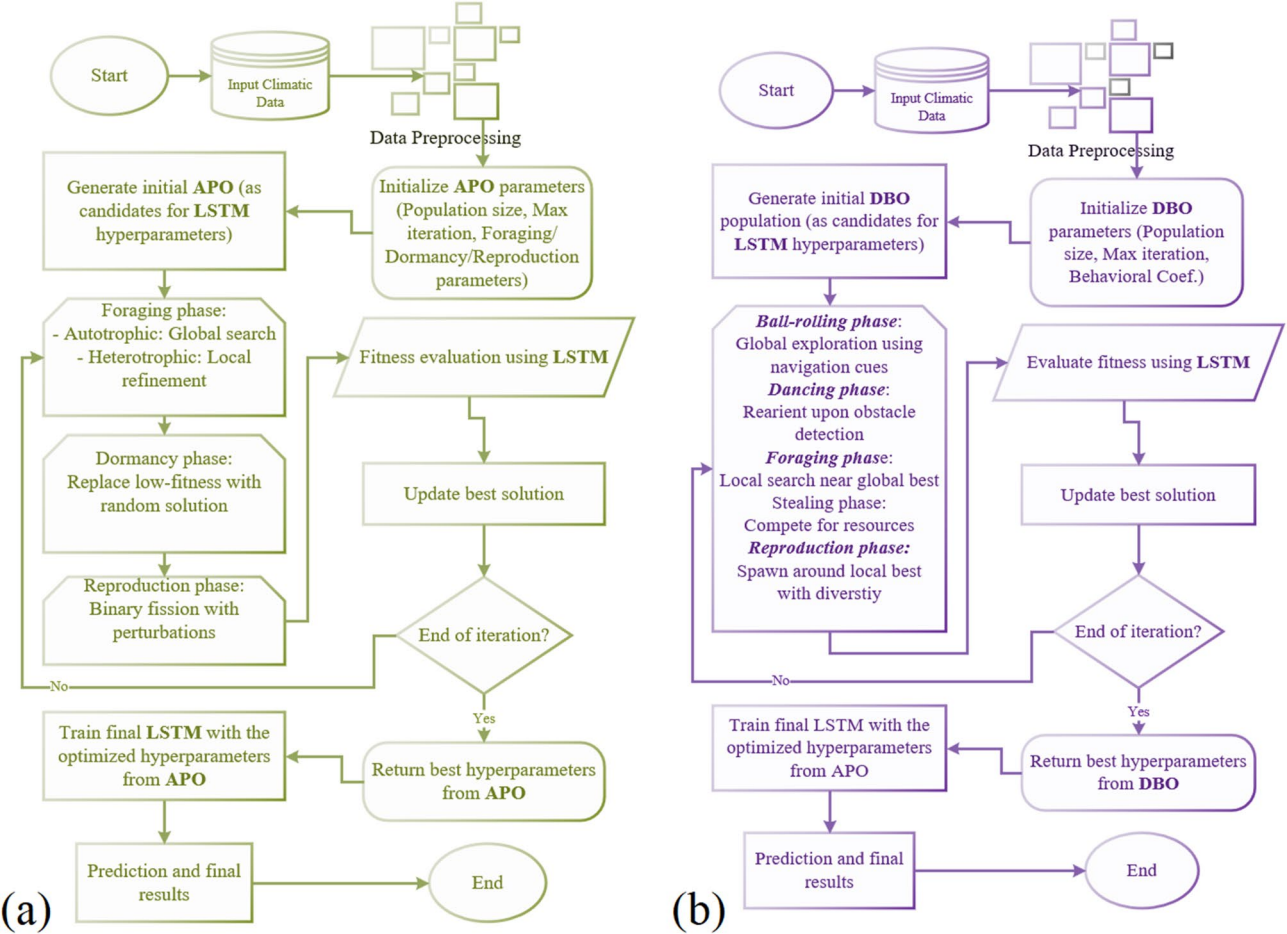
Schematic structure of **a** LSTM-APO and **b** LSTM-DBO models developed in this study.

Figure 8a depicts the general flowchart of the applied LSTM-APO procedure in this study. As can be seen in Fig. 8a, the optimization process in LSTM-APO begins with the initialization of a population of protozoa. Each agent represents a candidate set of LSTM hyperparameters. The APO, which in embedded with the LSTM, iteratively updates these solutions by simulating protozoan survival mechanisms. For this, autotrophic foraging guides the population toward promising regions by modulating movement intensity the (see Eq. 5). As this process happens, heterotrophic foraging refines local searches around rich areas of nutrients. Dormancy replaces suboptimal solutions with random ones to escape local optima, and reproduction introduces perturbations for fine-tuning. The fitness function, typically based on metrics like RMSE or NSE during training, evaluates each configuration, with the best hyperparameters selected to train the LSTM model on inputs variables (such as Tmin, Tmax, and Ra). The iterative process terminates after reaching a maximum number of evaluations or converging to an error threshold, yields an optimized LSTM mode for modeling evaporating. Computational workflow of the hybrid LSTM-APO model is given in the supplementary materials (Table S3).

#### Long Short Term Memory with Dung Beetle Optimizer (LSTM-DBO)

The Long Short-Term Memory with Dung Beetle Optimizer (LSTM-DBO) is another integrated model developed in this research. The structure of hybrid LSTM-DBO model proposed in this study is similar to the LSTM-APO model. As can be observed in Fig. 8b, adjusting the hyperparameters of the LSTM model is done by the application of the dung beetle behaviors such as ball-rolling for navigation, dancing for reorientation, foraging for resource acquisition, stealing for competition, and reproduction for population diversity. After setting up the initial population, the DBO divides its population into specialized roles including rolling beetles, brood balls, small beetles, and thieves to effectively balance global exploration and local exploitation. This integration allows DBO to optimize LSTM hyperparameters, including learning rate, number of hidden units, batch size, and dropout rate, addressing challenges like overfitting and suboptimal convergence in nonlinear evaporation processes with limited climatic inputs (e.g., Tmin, Tmax, Ra).

In the LSTM-DBO framework, the optimization initializes a population of dung beetles, each encoding a candidate LSTM hyperparameter set, and iteratively updates positions based on behavioral simulations. Ball-rolling updates, mimicking celestial navigation, are modeled (See Eq. 6). Fitness is assessed using metrics like RMSE during training on inputs variables (e.g., Tmin, Tmax, Ra). The optimization process would end after achieving the ideal residuals trough the training process or reaching maximum iterations. Computational workflow of the hybrid LSTM-DBO model is given in the supplementary materials (Table S4).

In this study, the optimization algorithms are employed to determine the optimal values of the LSTM hyperparameters. These algorithms search for the best combination of parameters by minimizing the objective function (RMSE). The optimized hyperparameter settings obtained using different algorithms are summarized in Table 2.

**Table 2:** Optimal hyperparameters of optimized LSTM models.

Parameter	Range	LSTM-GWO	LSTM-HHA	LSTM-DBO	LSTM-APO
Hidden units	20–200	120	110	90	70
Number of LSTM layers	1–3	2	1	2	2
Learning rate	0.0001–0.5	0.002	0.003	0.003	0.002
Batch size	16–256	64	32	32	64
Epochs	50–200	100	100	90	80
Dropout rate	0.05–0.6	0.20	0.15	0.20	0.25
Activation function	Tanh/Sigmoid	Tanh	Tanh	Tanh	Tanh
Optimizer	Adam/Adadelta	Adam	Adam	Adam	Adam
Loss function	RMSE				

## Results and discussion

### Results

The viability of APO and DBO-based deep learning method (LSTM-APO and LSTM-DBO) was assessed by comparing with standard LSTM and other hybrid LSTM methods (e.g., LSTM-GWO and LSTM-HHO) in predicting monthly pan evaporation (Epan) using limited inputs. Training and testing results of the LSTM-based methods in Epan prediction are summed up in Tables 3, 4, 5, 6 and 7 for the first station.

**Table 3:** Training and test statistics of the models for EPAN prediction—Station 1 (Model 1) LSTM.

Model inputs	Training period						**Test period**							
	RMSE	MAE	*R* ^2^		NSE		**RMSE**		**MAE**		**R** ^2^		**NSE**	
M1 (70%−30%)														
Tmin, Tmax	0.7196	0.5638	0.8482		0.8476		**0.8102** ^*^		**0.6028**		**0.8452**		**0.8437**	
Tmin, Ra	0.8757	0.6395	0.7794		0.7786		0.9483		0.6668		0.7708		0.7674	
Tmax, Ra	0.7608	0.5847	0.8338		0.8331		0.8358		0.6557		0.8206		0.8159	
Tmin, Tmax, Ra	0.7237	0.5584	0.8495		0.8482		0.8157		0.6345		0.8363		0.8314	
Tmin, Tmax, Ra, α	0.7994	0.5906	0.8395		0.8294		0.8125		0.6449		0.8167		0.8153	
M2 (75%−25%)														
Tmin, Tmax	0.6839	0.5126	0.8637		0.8622		0.7228		0.5845		0.8295		0.8248	
Tmin, Ra	0.8142	0.6048	0.7948		0.7942		0.8374		0.6558		0.7928		0.7869	
Tmax, Ra	0.7216	0.5332	0.8604		0.8573		0.7361		0.5916		0.8466		0.8457	
Tmin, Tmax, Ra	0.6547	0.5135	0.8625		0.8618		**0.6826**		**0.5307**		**0.8803**		**0.8676**	
Tmin, Tmax, Ra, α	0.6928	0.5258	0.8847		0.8754		0.7149		0.5338		0.8502		0.8484	
M3 (80%−20%)														
**Tmin**,** Tmax**	**0.5433**	**0.4234**	**0.9043**		**0.8984**		**0.5508**		**0.4279**		**0.9027**		**0.8979**	
Tmin, Ra	0.7314	0.5728	0.8252		0.8186		0.7584		0.5872		0.8083		0.8072	
Tmax, Ra	0.6106	0.4807	0.8804		0.8735		0.6438		0.5028		0.8627		0.8613	
Tmin, Tmax, Ra	0.5609	0.4348	0.8969		0.8935		0.5636		0.4556		0.8946		0.8922	
Tmin, Tmax, Ra, α	0.5528	0.4336	0.9008		0.8976		0.6338		0.4482		0.8973		0.8914	

^*^Bold numbers indicates the best value (minimum RMSE or MAE and maximum R^2^ or NSE).

**Table 4:** Training and test statistics of the models for EPAN prediction—Station 1 (Model 2) LSTM-GWO.

Model inputs	Training period				Test period			
	RMSE	MAE	*R* ^2^	NSE	RMSE	MAE	*R* ^2^	NSE
M1 (70%−30%)								
Tmin, Tmax	0.6803	0.5461	0.8906	0.8846	0.7136	0.5564	0.8604	0.8593
Tmin, Ra	0.7854	0.5832	0.8244	0.8225	0.9057	0.6243	0.7943	0.7894
Tmax, Ra	0.6838	0.5167	0.8675	0.8658	0.7504	0.5738	0.8584	0.8549
Tmin, Tmax, Ra	0.6716	0.5095	0.8772	0.8727	0.7403	0.5609	0.8567	0.8546
Tmin, Tmax, Ra, α	0.6846	0.5358	0.8858	0.8749	**0.6938** ^*^	**0.5573**	**0.8668**	**0.8593**
M2 (75%−25%)								
Tmin, Tmax	0.5464	0.4083	0.9063	0.8962	**0.6062**	**0.4863**	**0.8973**	**0.8924**
Tmin, Ra	0.7518	0.5446	0.8341	0.8303	0.7908	0.5875	0.8024	0.7983
Tmax, Ra	0.6683	0.5003	0.8704	0.8681	0.7036	0.5706	0.8496	0.8438
Tmin, Tmax, Ra	0.5659	0.4294	0.8846	0.8794	0.6685	0.4927	0.8785	0.8736
Tmin, Tmax, Ra, α	0.5907	0.4583	0.9158	0.9142	0.6204	0.4949	0.8947	0.8862
M3 (80%−20%)								
Tmin, Tmax	0.4738	0.3737	0.9281	0.9257	0.5493	0.4317	0.9083	0.8993
Tmin, Ra	0.7166	0.5526	0.8253	0.8136	0.7436	0.5869	0.8191	0.8165
Tmax, Ra	0.5753	0.4535	0.8948	0.8741	0.6194	0.4954	0.8864	0.8834
**Tmin**,** Tmax**,** Ra**	**0.4828**	**0.3654**	**0.9266**	**0.9228**	**0.4892**	**0.3748**	**0.9246**	**0.9192**
Tmin, Tmax, Ra, α	0.5104	0.4042	0.9165	0.9132	0.5403	0.4253	0.9089	0.9056

^*^Bold numbers indicates the best value (minimum RMSE or MAE and maximum R^2^ or NSE).

**Table 5:** Training and test statistics of the models for EPAN prediction—Station 1 (Model 3) LSTM-HHO.

Model inputs	Training period				Test period				
	RMSE	MAE	*R* ^2^	NSE	RMSE		MAE	*R* ^2^	NSE
M1 (70%−30%)									
Tmin, Tmax	0.6348	0.4938	0.8871	0.8848	0.7857		0.6152	0.8607	0.8469
Tmin, Ra	0.8331	0.6152	0.7996	0.7987	0.9224		0.6364	0.7829	0.7803
Tmax, Ra	0.6305	0.4822	0.8862	0.8853	0.7602		0.5365	0.8583	0.8524
Tmin, Tmax, Ra	0.6347	0.4959	0.8874	0.8852	**0.7418** ^*^		**0.5903**	**0.8671**	**0.8631**
Tmin, Tmax, Ra, α	0.6322	0.4914	0.8868	0.8851	0.7853		0.6256	0.8596	0.8528
M2 (75%−25%)									
Tmin, Tmax	0.5673	0.4223	0.9063	0.9046	**0.5857**		**0.4557**	**0.9024**	**0.8982**
Tmin, Ra	0.7801	0.5628	0.8221	0.8208	0.7883		0.5793	0.8068	0.8013
Tmax, Ra	0.6406	0.4657	0.8796	0.8783	0.6868		0.5242	0.8583	0.8492
Tmin, Tmax, Ra	0.5502	0.4452	0.8992	0.8974	0.5882		0.4654	0.8982	0.8961
Tmin, Tmax, Ra, α	0.5734	0.4554	0.9178	0.8994	0.5934		0.4748	0.8944	0.8926
M3 (80%−20%)									
Tmin, Tmax	0.4451	0.3482	0.9362	0.9344	0.5048		0.4004	0.9293	0.9272
Tmin, Ra	0.7067	0.5574	0.8308	0.8282	0.7452		0.5708	0.8264	0.8241
Tmax, Ra	0.5258	0.4161	0.9132	0.9083	0.6223		0.4853	0.8921	0.8904
Tmin, Tmax, Ra	0.4668	0.3704	0.9344	0.9292	0.5131		0.4112	0.9134	0.9121
**Tmin**,** Tmax**,** Ra**,** α**	**0.4103**	**0.3226**	**0.9458**	**0.9437**	**0.4614**		**0.3621**	**0.9384**	**0.9348**

^*^Bold numbers indicates the best value (minimum RMSE or MAE and maximum R^2^ or NSE).

**Table 6:** Training and test statistics of the models for EPAN prediction—Station 1 (Model 4) LSTM-DBO.

Model inputs	Training period				Test period			
	RMSE	MAE	*R* ^2^	NSE	RMSE	MAE	*R* ^2^	NSE
M1 (70%−30%)								
Tmin, Tmax	0.6503	0.5161	0.8952	0.8881	0.6957	0.5702	0.8792	0.8781
Tmin, Ra	0.8204	0.5904	0.8144	0.8124	0.8534	0.6003	0.8084	0.8063
Tmax, Ra	0.6567	0.5052	0.8776	0.8758	0.7902	0.5741	0.8423	0.8392
Tmin, Tmax, Ra	0.5802	0.4448	0.9041	0.9013	**0.6753** ^*^	**0.5542**	**0.8841**	**0.8810**
Tmin, Tmax, Ra, α	0.5974	0.4836	0.9004	0.8982	0.7961	0.6204	0.8458	0.8396
M2 (75%−25%)								
Tmin, Tmax	0.5424	0.3952	0.9161	0.9144	**0.5953**	**0.4657**	**0.9106**	**0.8992**
Tmin, Ra	0.7578	0.5518	0.8308	0.8295	0.7876	0.5956	0.8034	0.7992
Tmax, Ra	0.6253	0.4667	0.8882	0.8854	0.6656	0.5302	0.8682	0.8614
Tmin, Tmax, Ra	0.5367	0.3859	0.9163	0.9146	0.6042	0.4838	0.9035	0.8946
Tmin, Tmax, Ra, α	0.6131	0.4773	0.8992	0.8978	0.6403	0.5057	0.8784	0.8713
M3 (80%−20%)								
Tmin, Tmax	0.5051	0.4327	0.8996	0.8991	0.5490	0.3942	0.9261	0.9208
Tmin, Ra	0.7128	0.5553	0.8324	0.8308	0.7257	0.5618	0.8293	0.8236
Tmax, Ra	0.5062	0.3934	0.9252	0.9208	0.5953	0.4502	0.8831	0.8824
Tmin, Tmax, Ra	0.4859	0.3851	0.9295	0.9267	0.5067	0.3938	0.9398	0.9376
**Tmin**,** Tmax**,** Ra**,** α**	**0.4063**	**0.3293**	**0.9538**	**0.9493**	**0.4452**	**0.3402**	**0.9475**	**0.9442**

^*^Bold numbers indicates the best value (minimum RMSE or MAE and maximum R^2^ or NSE).

**Table 7:** Training and test statistics of the models for EPAN prediction—Station 1 (Model 5) LSTM-APO.

Model inputs	Training period				Test period			
	RMSE	MAE	*R* ^2^	NSE	RMSE	MAE	*R* ^2^	NSE
M1 (70%−30%)								
Tmin, Tmax	0.5073	0.3928	0.9272	0.9256	0.6408	0.5154	0.9134	0.9014
Tmin, Ra	0.7404	0.5376	0.8434	0.8418	0.8353	0.5802	0.8221	0.8196
Tmax, Ra	0.5008	0.3828	0.9284	0.9273	0.6116	0.4856	0.9128	0.9084
Tmin, Tmax, Ra	0.4603	0.3603	0.9402	0.9391	**0.6059** ^*^	**0.4691**	**0.9255**	**0.9168**
Tmin, Tmax, Ra, α	0.4418	0.3454	0.9466	0.9442	0.6172	0.5138	0.9208	0.9143
M2 (75%−25%)								
Tmin, Tmax	0.4326	0.3156	0.9462	0.9451	0.5406	0.4114	0.9436	0.9228
Tmin, Ra	0.6982	0.5103	0.8573	0.8556	0.7867	0.5902	0.8054	0.7993
Tmax, Ra	0.4604	0.3417	0.9396	0.9382	0.5738	0.4357	0.9143	0.8994
Tmin, Tmax, Ra	0.3901	0.2828	0.9568	0.9553	**0.5164**	**0.3932**	**0.9496**	**0.9264**
Tmin, Tmax, Ra, α	0.3928	0.2896	0.9554	0.9538	0.5209	0.4028	0.9484	0.9206
M3 (80%−20%)								
**Tmin**,** Tmax**	**0.3623**	**0.2783**	**0.9592**	**0.9573**	**0.2954**	**0.2256**	**0.9763**	**0.9714**
Tmin, Ra	0.6781	0.5176	0.8473	0.8456	0.6803	0.5352	0.8496	0.8437
Tmax, Ra	0.4408	0.3372	0.9371	0.9354	0.4428	0.3478	0.9408	0.9368
Tmin, Tmax, Ra	0.3304	0.2564	0.9654	0.9642	0.3456	0.2743	0.9656	0.9604
Tmin, Tmax, Ra, α	0.3317	0.2601	0.9646	0.9628	0.3853	0.3127	0.9573	0.9525

^*^Bold numbers indicates the best value (minimum RMSE or MAE and maximum R^2^ or NSE).

As evident from Table 4 that the training accuracy of standard LSTM model range 0.719 to 0.876 mm for M1, 0.684 to 0.814 mm for M2 and 0.543 to 0.731 mm for M3 with respect to RMSE while the corresponding testing ranges are 0.810–0.948 mm, 0.683–0.837 mm, and 0.551–0.758 mm for M1, M2 and M3, respectively. The LSTM offers the best accuracy with Tmin and Tmax inputs (input combination 1) for M1 and M3 data sets while the 4th input combination (Tmin, Tmax, Ra) provides the best predictions for M2 data set in the testing stage. Overall, the best Epan prediction are obtained from the M3 data set with the lowest RMSE (0.551 mm), MAE (0.428 mm) and the highest NSE (0.898).

It is clear from Tables 4, 5, 6 and 7 that the hybrid LSTM methods also offer the best accuracy for the M3 testing data set as found for the standard LSTM method. For this testing data set, the training accuracy of LSTM-GWO, LSTM-HHO, LSTM-SHO and LSTM-ESHO ranges 0.474–0.717 mm, 0.410–0.707 mm, 0.406–0.713 mm, and 0.330–0.441 mm, respectively while their corresponding ranges in the testing stage are 0.489–0.744 mm, 0.461–0.801 mm, 0.445–0.725 mm, and 0.295–0.443 mm with respect to RMSE. It is clear from the ranges that the hybrid LSTM methods improve the Epan prediction accuracy compared to standard LSTM method. For example, the best LSTM-APO model (M3 case) improved the RMSE, MAE, R², and NSE from 0.551 mm, 0.428 mm, 0.903, and 0.898 (standard LSTM) to 0.295 mm, 0.226 mm, 0.976, and 0.971 in the testing stage, respectively. The LSTM-DBO also showed notable improvements, achieving RMSE = 0.445 mm, MAE = 0.340 mm, R² = 0.948, and NSE = 0.944 for the same data set.

The best input combinations differ with respect to testing data set (e.g., M1, M2, M3) when applying hybrid LSTM methods. For example, LSTM-GWO with 4th input combination (Tmin, Tmax, Ra) has the best accuracy for the M2 and M3 data sets while the 1 st (Tmin, Tmax) offers the best accuracy in testing stage of M1 data set. Similarly, the best input combinations for LSTM-HHO were the 4th, 4th, and 4th for M1, M2, and M3, respectively. For LSTM-DBO, the best accuracy was obtained with the 3rd, 4th, and 5th input combinations in M1, M2, and M3, respectively. In the case of LSTM-APO, the best predictions were produced by the 3rd input combination for M1, the 4th input combination for M2, and the 1 st input combination for M3.

Training and testing outcomes of the LSTM-based methods in predicting Epan of second station are listed in Tables 8, 9, 10, 11 and 12. Unlike the first station, here standard LSTM method has the best accuracy in predicting Epan for M2 data set. Training and testing accuracy of the LSTM for this data set ranges 0.763–0.904 mm and 0.774–0.917 mm. The best input combination belongs to 3rd, 1 st and 1 st input combinations for the M1, M2 and M3 testing data sets, respectively. A similar observation exists for the hybrid LSTM-GWO and LSTM-HHO methods as evident from Tables 9 and 10. However, the best accuracy of the LSTM-DBO was obtained with the 3rd, 4th, and 5th input combinations for M1, M2, and M3, respectively, while the LSTM-APO produced its best performance with the 2nd, 4th, and 1 st input combinations in the testing stage.

**Table 8:** Training and test statistics of the models for EPAN prediction—Station 2 (Model 1) LSTM.

Model inputs	Training period				Test period			
	RMSE	MAE	*R* ^2^	NSE	RMSE	MAE	*R* ^2^	NSE
M1 (70%−30%)								
Tmin, Tmax	0.9406	0.6636	0.8703	0.8668	**0.9186** ^*^	**0.7043**	**0.8302**	**0.8273**
Tmin, Ra	0.9738	0.7324	0.8016	0.8004	0.9982	0.7756	0.8036	0.7982
Tmax, Ra	0.9067	0.6451	0.8328	0.8293	0.9454	0.6508	0.8274	0.8242
Tmin, Tmax, Ra	0.9504	0.7208	0.8304	0.8264	0.9946	0.7304	0.8098	0.8055
Tmin, Tmax, Ra, α	0.9535	0.7067	0.8229	0.8201	0.9848	0.7126	0.8064	0.8048
M2 (75%−25%)								
**Tmin**,** Tmax**	**0.7627**	**0.5816**	**0.8934**	**0.8872**	**0.7738**	**0.5802**	**0.8712**	**0.8683**
Tmin, Ra	0.9038	0.7015	0.8256	0.8224	0.9142	0.7064	0.8234	0.8191
Tmax, Ra	0.8341	0.6332	0.8492	0.8441	0.8937	0.6728	0.8338	0.8308
Tmin, Tmax, Ra	0.8688	0.6728	0.8371	0.8328	0.8992	0.6803	0.8302	0.8276
Tmin, Tmax, Ra, α	0.8606	0.6414	0.8453	0.8437	0.8657	0.6693	0.8381	0.8352
M3 (80%−20%)								
Tmin, Tmax	0.8238	0.6368	0.8692	0.8628	**0.8654**	**0.6878**	**0.8643**	**0.8618**
Tmin, Ra	0.9204	0.7093	0.8164	0.8143	0.9963	0.7982	0.7918	0.7867
Tmax, Ra	0.8517	0.6727	0.8508	0.8466	0.9914	0.8104	0.8035	0.7982
Tmin, Tmax, Ra	0.8806	0.6764	0.8303	0.8291	0.9997	0.8163	0.8016	0.7996
Tmin, Tmax, Ra, α	0.8704	0.6639	0.8351	0.8337	0.9804	0.7904	0.8153	0.8114

^*^Bold numbers indicates the best value (minimum RMSE or MAE and maximum R^2^ or NSE).

**Table 9:** Training and test statistics of the models for EPAN prediction—Station 2 (Model 2) LSTM-GWO.

Model inputs	Training period				Test period			
	RMSE	MAE	*R* ^2^	NSE	RMSE	MAE	*R* ^2^	NSE
M1 (70%−30%)								
Tmin, Tmax	0.8764	0.6674	0.8483	0.8436	0.9689	0.6708	0.8435	0.8404
Tmin, Ra	0.8993	0.6678	0.8568	0.8516	0.9042	0.6816	0.8336	0.8302
Tmax, Ra	0.8104	0.5973	0.8715	0.8673	0.8408	0.6179	0.8672	0.8642
Tmin, Tmax, Ra	0.8235	0.6303	0.8578	0.8528	0.8607	0.6326	0.8508	0.8468
Tmin, Tmax, Ra, α	0.7726	0.5838	0.8873	0.8828	**0.8193** ^*^	**0.6018**	**0.8793**	**0.8738**
M2 (75%−25%)								
**Tmin**,** Tmax**	**0.6808**	**0.5117**	**0.8997**	**0.8972**	**0.6836**	**0.5304**	**0.9017**	**0.8973**
Tmin, Ra	0.7703	0.5852	0.8702	0.8678	0.7928	0.6064	0.8653	0.8628
Tmax, Ra	0.7064	0.5338	0.8915	0.8894	0.7204	0.5652	0.8891	0.8862
Tmin, Tmax, Ra	0.7137	0.5659	0.8936	0.8904	0.7336	0.5737	0.8824	0.8796
Tmin, Tmax, Ra, α	0.7608	0.5946	0.8752	0.8716	0.8108	0.6039	0.8592	0.8564
M3 (80%−20%)								
Tmin, Tmax	0.7038	0.5064	0.8996	0.8968	**0.7773**	**0.5934**	**0.8852**	**0.8814**
Tmin, Ra	0.8258	0.6294	0.8578	0.8553	0.8708	0.6672	0.8416	0.8373
Tmax, Ra	0.8207	0.6303	0.8592	0.8578	0.8973	0.7258	0.8364	0.8328
Tmin, Tmax, Ra	0.8982	0.7052	0.8396	0.8342	0.9602	0.7629	0.8268	0.8189
Tmin, Tmax, Ra, α	0.6851	0.5424	0.9056	0.9028	0.8027	0.6707	0.8816	0.8618

^*^Bold numbers indicates the best value (minimum RMSE or MAE and maximum R^2^ or NSE).

**Table 10:** Training and test statistics of the models for EPAN prediction—Station 2 (Model 3) LSTM-HHO.

Model inputs	Training period				Test period			
	RMSE	MAE	*R* ^2^	NSE	RMSE	MAE	*R* ^2^	NSE
M1								
Tmin, Tmax	0.8083	0.6173	0.8993	0.8934	0.8453	0.6382	0.8752	0.8613
Tmin, Ra	0.8536	0.6608	0.8496	0.8482	0.9358	0.6658	0.8473	0.8428
Tmax, Ra	0.8364	0.7056	0.8604	0.8568	0.8867	0.7303	0.8542	0.8512
Tmin, Tmax, Ra	0.7608	0.5804	0.8712	0.8695	**0.8326** ^*^	**0.5956**	**0.8979**	**0.8936**
Tmin, Tmax, Ra, α	0.8202	0.6852	0.8255	0.8234	0.8543	0.6957	0.8204	0.8173
M2								
Tmin, Tmax	0.6716	0.5203	0.9028	0.9006	0.7304	0.5564	0.8834	0.8816
Tmin, Ra	0.8572	0.6704	0.8526	0.8492	0.7356	0.5856	0.8806	0.8793
**Tmax**,** Ra**	0.6906	0.5407	0.9021	0.8993	0.8208	0.6452	0.8542	0.8508
Tmin, Tmax, Ra	0.7864	0.6106	0.8968	0.8928	0.8473	0.6737	0.8753	0.8713
Tmin, Tmax, Ra, α	**0.6528**	**0.4938**	**0.9138**	**0.9124**	**0.6824**	**0.5382**	**0.9045**	**0.8998**
M3								
Tmin, Tmax	0.6843	0.4929	0.8983	0.8972	**0.7808**	**0.6226**	**0.8893**	**0.8852**
Tmin, Ra	0.7608	0.6013	0.8758	0.8718	0.8679	0.6468	0.8404	0.8378
Tmax, Ra	0.7293	0.5658	0.8862	0.8834	0.8004	0.6193	0.8781	0.8726
Tmin, Tmax, Ra	0.7116	0.5336	0.8954	0.8893	0.9193	0.7247	0.8596	0.8558
Tmin, Tmax, Ra, α	0.7858	0.6057	0.8667	0.8639	0.8186	0.6652	0.8515	0.8502

^*^Bold numbers indicates the best value (minimum RMSE or MAE and maximum R^2^ or NSE).

**Table 11:** Training and test statistics of the models for EPAN prediction—Station 2 (Model 4) LSTM-DBO.

Model inputs	Training period				Test period				
	RMSE	MAE	*R* ^2^	NSE	RMSE		MAE	*R* ^2^	NSE
M1 (70%−30%)									
Tmin, Tmax	0.7476	0.5542	0.8852	0.8841	0.9281		0.7063	0.8482	0.8436
Tmin, Ra	0.7514	0.5861	0.8836	0.8831	0.8703		0.6692	0.8653	0.8614
Tmax, Ra	0.7973	0.6004	0.8728	0.8692	0.8326		0.6154	0.8685	0.8647
Tmin, Tmax, Ra	0.7708	0.5605	0.8784	0.8775	0.8242		0.6038	0.8738	0.8706
Tmin, Tmax, Ra, α	0.6972	0.5482	0.9026	0.8994	**0.8058**		**0.5985**	**0.8804**	**0.8728**
M2 (75%−25%)									
**Tmin**,** Tmax**	**0.4863** ^*^	**0.3808**	**0.9492**	**0.9481**	**0.5293**		**0.4406**	**0.9382**	**0.9346**
Tmin, Ra	0.7828	0.5806	0.8674	0.8656	0.8106		0.6258	0.8549	0.8535
Tmax, Ra	0.6553	0.5046	0.9076	0.9058	0.6584		0.5137	0.9038	0.9026
Tmin, Tmax, Ra	0.5167	0.4608	0.9385	0.9346	0.6708		0.4934	0.9156	0.9128
Tmin, Tmax, Ra, α	0.6309	0.5237	0.9003	0.8982	0.6839		0.5395	0.9064	0.9053
M3 (80%−20%)									
Tmin, Tmax	0.6708	0.5356	0.9082	0.9061	0.7682		0.6144	0.8904	0.8868
Tmin, Ra	0.7136	0.5659	0.8934	0.8928	0.9236		0.7303	0.8328	0.8304
Tmax, Ra	0.8594	0.6508	0.8456	0.8449	0.9674		0.7738	0.8126	0.8089
Tmin, Tmax, Ra	0.7056	0.5337	0.8967	0.8953	0.8438		0.6356	0.8703	0.8664
Tmin, Tmax, Ra, α	0.6608	0.5205	0.9092	0.9081	**0.7596**		**0.6019**	**0.8924**	**0.8896**

^*^Bold numbers indicates the best value (minimum RMSE or MAE and maximum R^2^ or NSE).

**Table 12:** Training and test statistics of the models for EPAN prediction – Station 2 (Model 5) LSTM-APO.

Model inputs	Training period				Test period				
	RMSE	MAE	*R* ^2^	NSE	RMSE		MAE	*R* ^2^	NSE
M1 (70%−30%)									
Tmin, Tmax	0.6034	0.4703	0.9263	0.9252	0.7772		0.6082	0.9391	0.9356
Tmin, Ra	0.6886	0.5368	0.9052	0.9038	0.7578		0.5774	0.9028	0.8996
Tmax, Ra	0.6395	0.4857	0.9168	0.9152	0.7476		0.5653	0.9093	0.9054
Tmin, Tmax, Ra	0.5682	0.4354	0.9346	0.9334	**0.7269** ^*^		**0.5591**	**0.9276**	**0.9244**
Tmin, Tmax, Ra, α	0.7629	0.6309	0.8474	0.8452	0.8358		0.5858	0.8392	0.8358
M2 (75%−25%)									
**Tmin**,** Tmax**	**0.4059**	**0.3154**	**0.9738**	**0.9706**	**0.4338**		**0.3454**	**0.9606**	**0.9614**
Tmin, Ra	0.6868	0.5267	0.8976	0.8961	0.7036		0.5506	0.8908	0.8893
Tmax, Ra	0.5727	0.4368	0.9319	0.9293	0.5894		0.4608	0.9284	0.9256
Tmin, Tmax, Ra	0.4804	0.3757	0.9514	0.9502	0.5299		0.4219	0.9439	0.9405
Tmin, Tmax, Ra, α	0.6583	0.5129	0.9073	0.9056	0.7336		0.5637	0.8827	0.8796
M3 (80%−20%)									
Tmin, Tmax	0.5258	0.4046	0.9438	0.9426	0.7108		0.5807	0.9124	0.9096
Tmin, Ra	0.6479	0.5108	0.9136	0.9124	0.7654		0.5928	0.8774	0.8743
Tmax, Ra	0.5726	0.4429	0.9325	0.9313	0.7306		0.5636	0.9182	0.9145
Tmin, Tmax, Ra	0.5052	0.3951	0.9489	0.9475	0.6819		0.5654	0.9246	0.9208
Tmin, Tmax, Ra, α	0.4708	0.3664	0.9554	0.9541	**0.6725**		**0.5538**	**0.9328**	**0.9282**

^*^Bold numbers indicates the best value (minimum RMSE or MAE and maximum R^2^ or NSE).

Tables 9, 10, 11 and 12 clearly illustrate that the hybrid LSTM methods also have the best accuracy in predicting Epan of the 2nd station for M2 data set as found for the standard LSTM. For the M2 data set, the accuracy of LSTM-GWO, LSTM-HHO, LSTM-SHO and LSTM-ESHO in training stage ranges 0.681 to 0.899 mm, 0.652 to 0.857 mm, 0.517 to 0.783 mm, and 0.406 to 0.698 mm, respectively while their corresponding ranges in the testing stage are 0.684–0.901 mm, 0.682–0.821 mm, 0.529–0.811 mm and 0.434–0.734 mm with respect to RMSE. It is clearly seen from the ranges the prediction accuracy is improved by employing hybrid LSTM methods. For example, the LSTM-APO model (M2 case) decreased RMSE and MAE from 0.774 mm to 0.582 mm (standard LSTM) to 0.434 mm and 0.345 mm, respectively, while increasing R² and NSE from 0.869 to 0.868 to 0.961 and 0.961. Similarly, the LSTM-DBO model improved the prediction accuracy by reducing RMSE to 0.529 mm and raising NSE to 0.935. These results confirm that APO and DBO considerably enhance the predictive power and robustness of LSTM compared to both the standard LSTM and the conventional hybrid approaches.

**Fig. 9:**
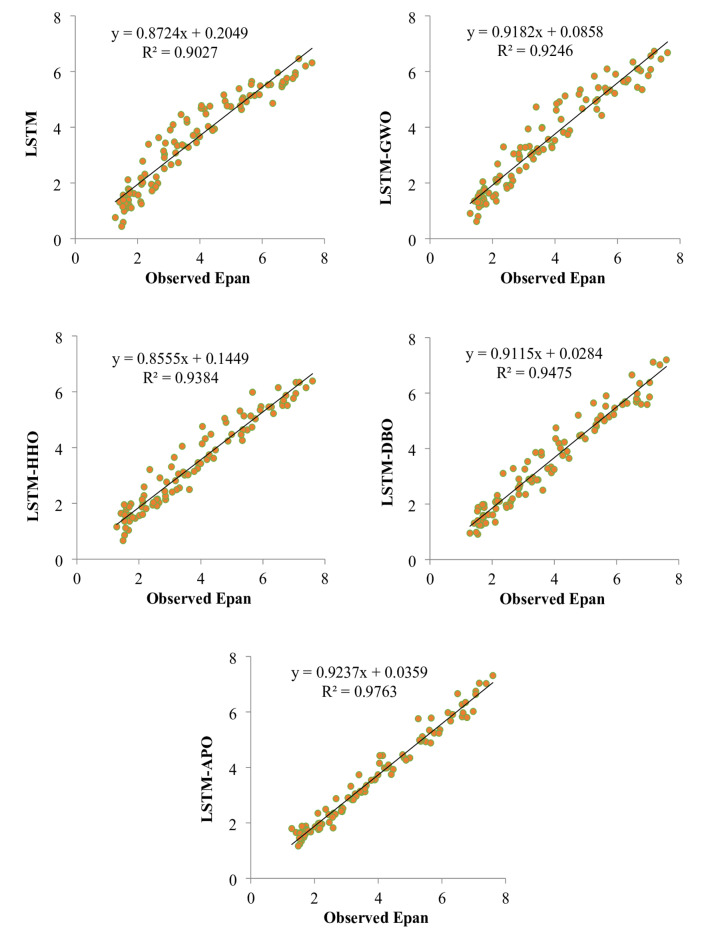
Scatterplots of the observed and predicted Epan by different LSTM based models in the test period using best input combination—Station 1.

**Fig. 10:**
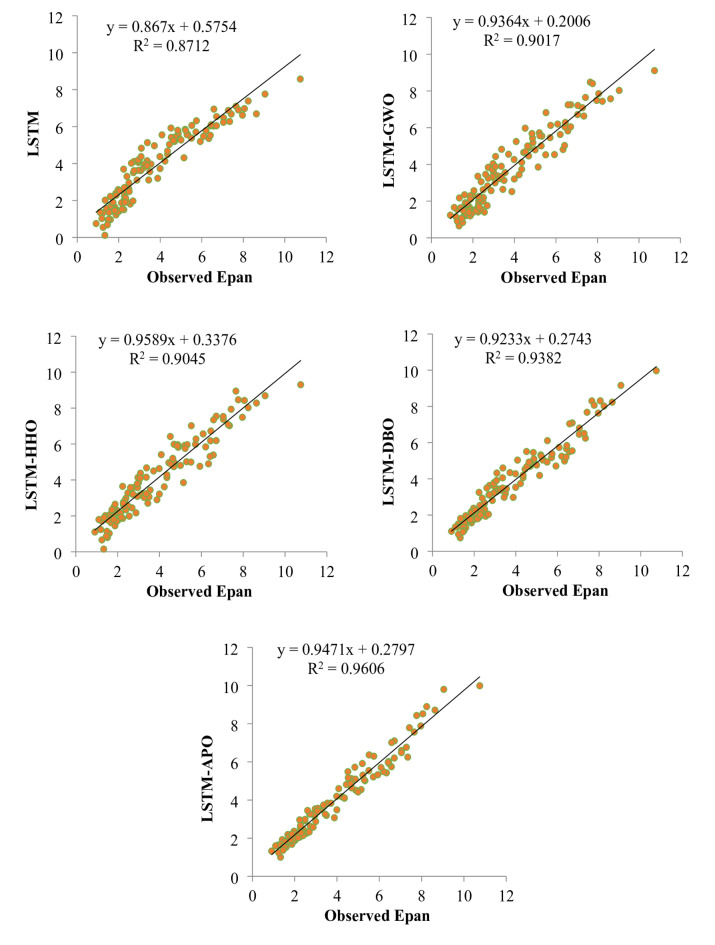
Scatterplots of the observed and predicted Epan by different LSTM based models in the test period using best input combination—Station 2.

**Fig. 11:**
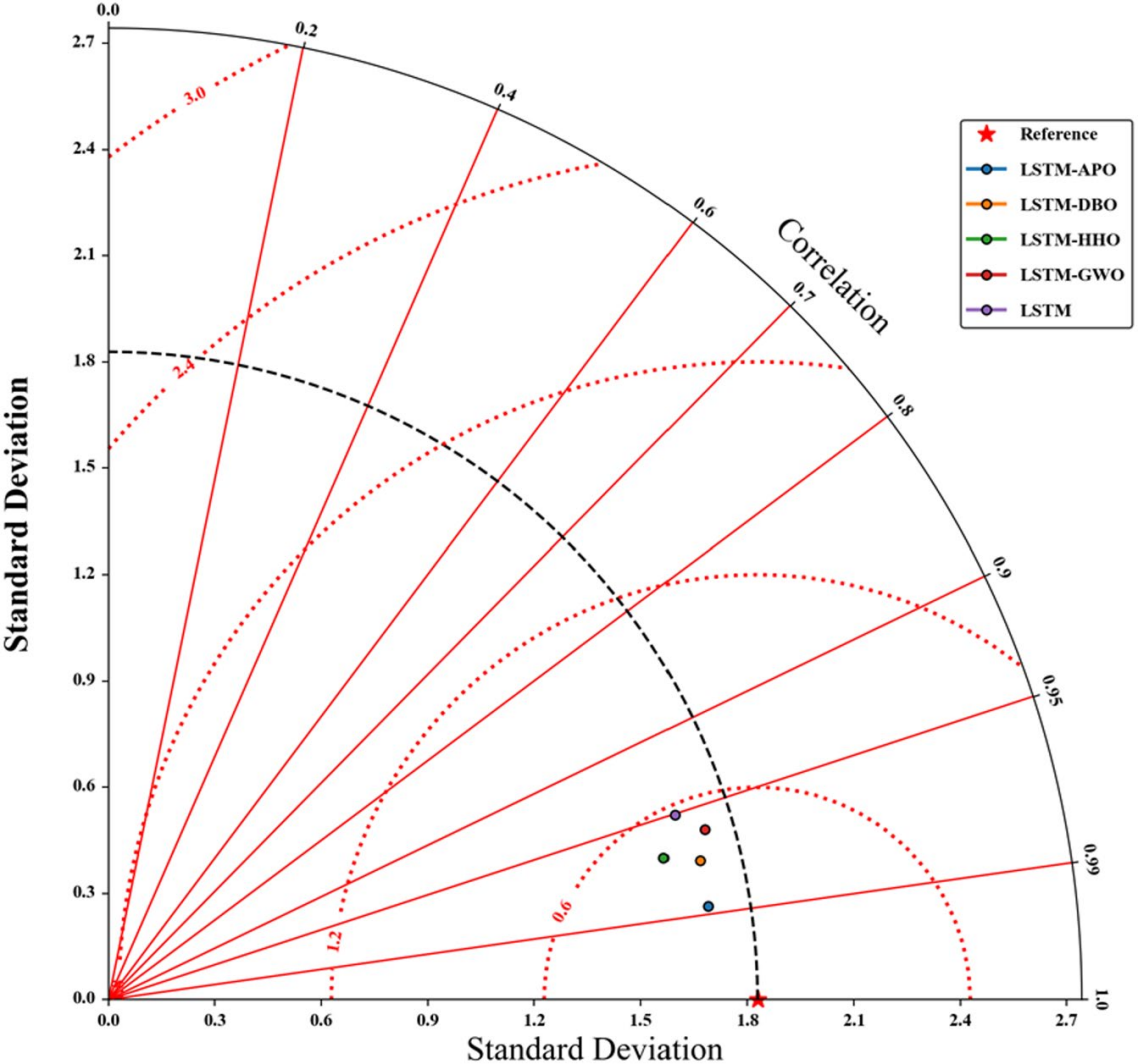
Taylor diagrams of the observed and predicted Epan by different LSTM based models in the test period using the best input combination—Station 1.

**Fig. 12:**
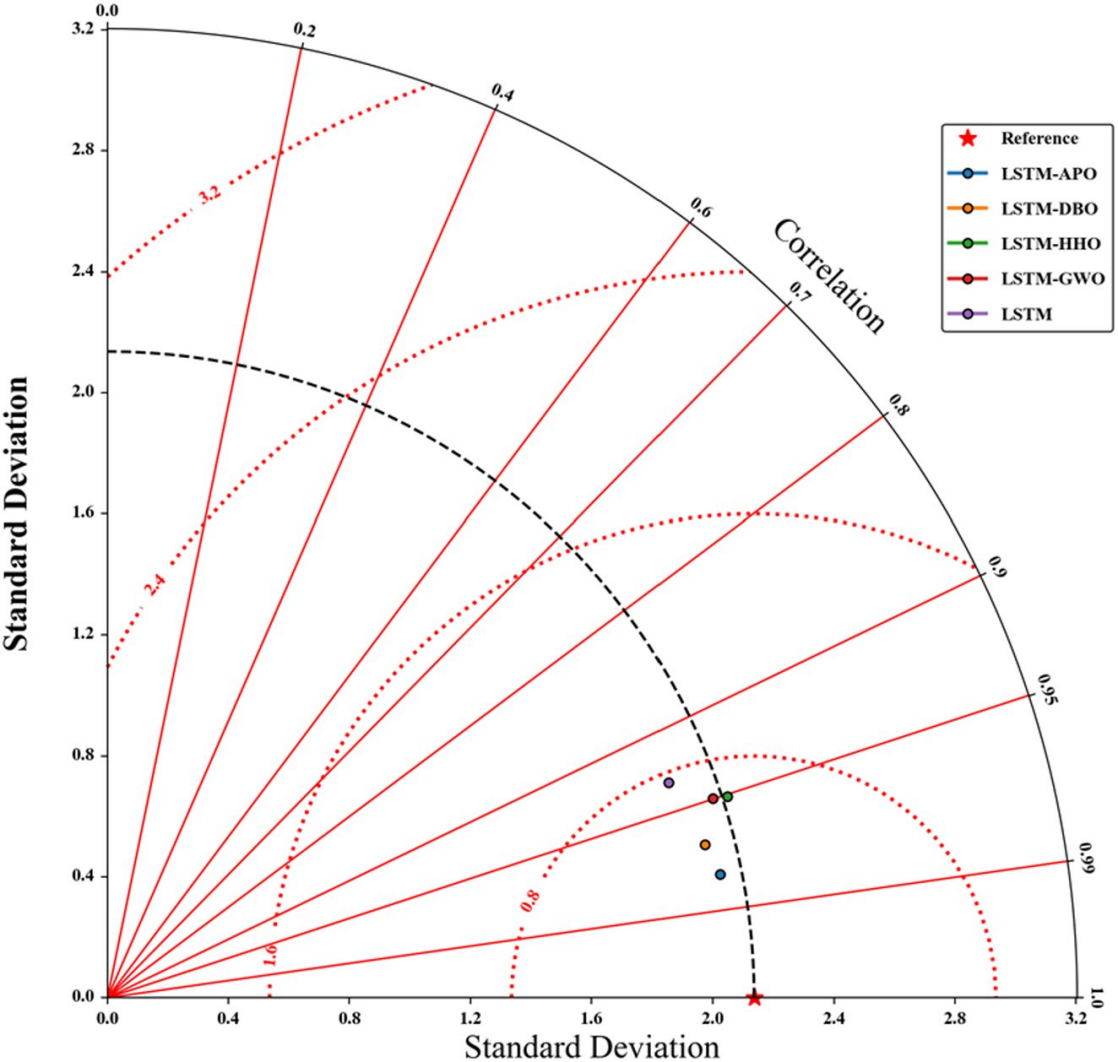
Taylor diagrams of the observed and predicted Epan by different LSTM based models in the test period using the best input combination—Station 2.

**Fig. 13:**
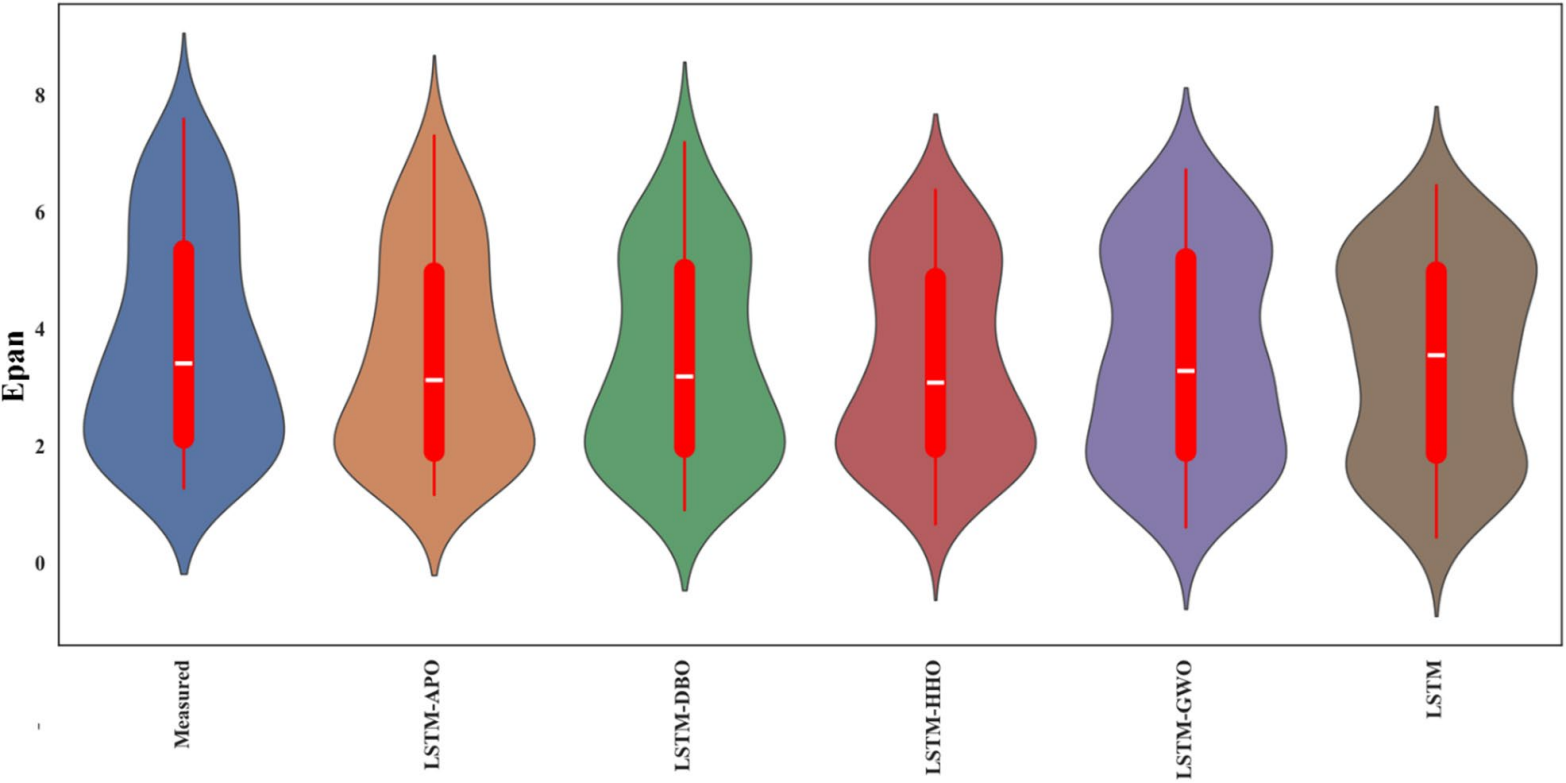
Violin charts of the observed and predicted Epan by different LSTM based models in the test period using the best input combination—Station 1.

**Fig. 14:**
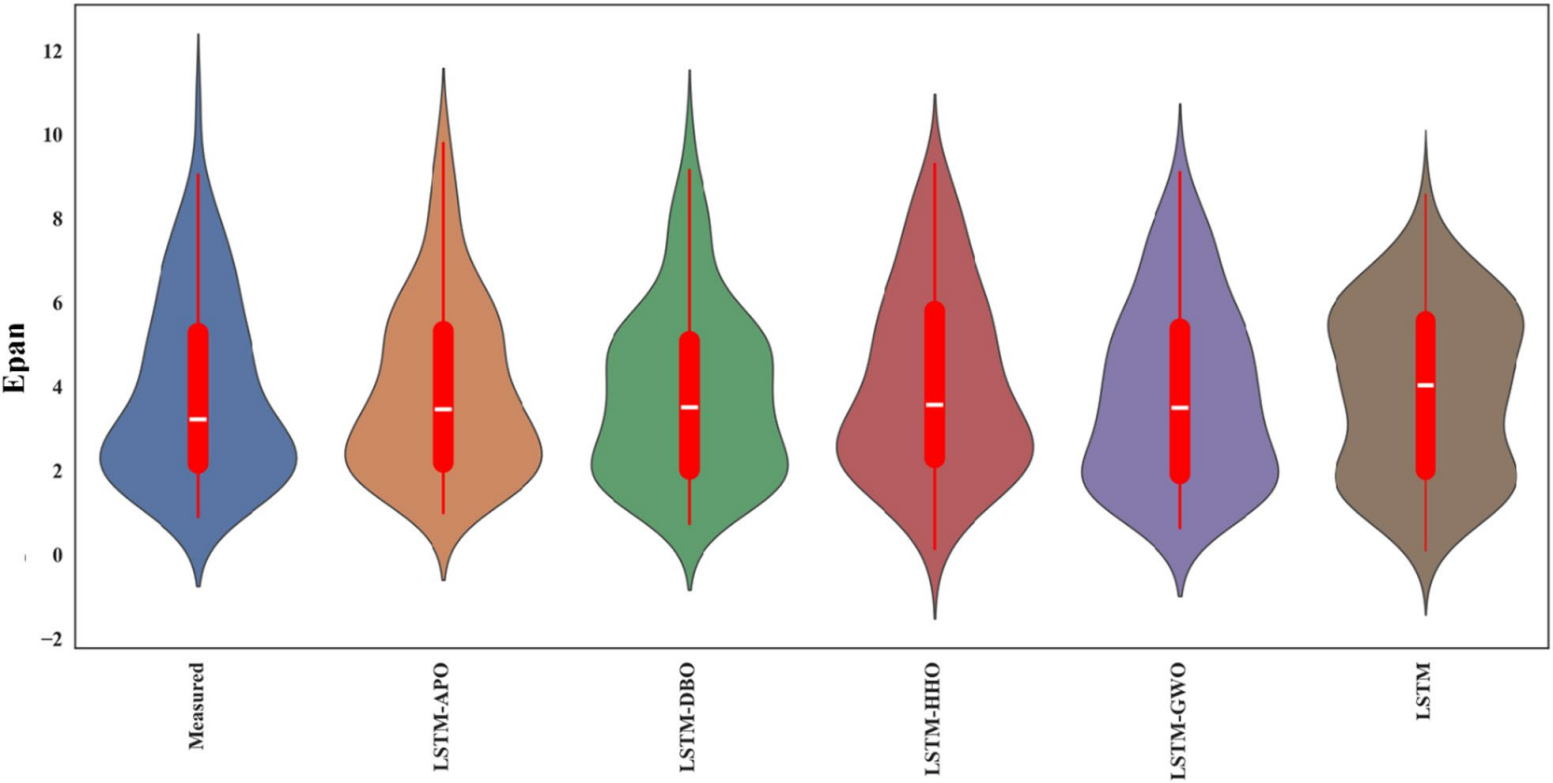
Violin charts of the observed and predicted Epan by different LSTM based models in the test period using the best input combination—Station 2.

Figures 9 and 10 depict scatterplots comparing the observed and predicted pan evaporation at two stations in the test period. The scatter graphs show that the LSTM-APO model produces more accurate predictions with less variability compared to other models, closely followed by the LSTM-DBO model. The fitted line equations reveal that the slopes and biases of these proposed models align more closely with the ideal line y = x than the LSTM and other hybrid models. To comprehensively assess the LSSTM-based models, Figs. 11 and 12 represent Taylor diagrams for the test period, allowing for the simultaneous evaluation of multiple statistics. It is clearly appeared from the figures that the LSTM-APO model provides the lowest RMSE, the highest correlation, and a standard deviation closely aligns with the observed values in both stations, with LSTM-DBO also performing competetitively. Additionally, Figs. 13 and 14 display violin charts providing further insight into the distribution of predicted pan evaporation by the hybrid models compared to the observed values. These charts illustrate that the distribution obtained by LSTM-APO most closely mirrors the observed distribution, while the LSTM-DBO also demonstrates consistent performance. Furthermore, the graphs distinctly show that incorporating metaheuristic algorithms such as APO and DBO considerably enhances the predictive accuracy of the standard LSTM model.

### Discussion

The capability of LSTM-based hybrid methods were tested in predicting Epan and compared with the standard LSTM. It was found that merging metaheuristic algorithms such as GWO, HHO, APO and DBO considerably improves the accuracy of the standard LSTM in EPan prediction using limited inputs. By implementing the LSTM-APO, the RMSE, MAE, R^2^ and NSE of the best LSTM model (M3 case, Station 1) in the testing stage improved by 46.5%, 47.2%, 7.3%, and 8.1%. Similarly, the LSTM-APO in Station 2 (M2 case) reduced the RMSE and MAE by 43.9% and 40.7%, while increasing R² and NSE by about 9%. The LSTM-DBO model also demonstrated remarkable improvements: in Station 1 (M3 case), RMSE decreased by nearly 19% compared to the standard LSTM, and in Station 2 (M2 case), RMSE and MAE values were reduced by 31.6% and 40.2%, respectively. Among all hybrid methods, the LSTM-APO offered the highest accuracy, followed by LSTM-DBO, indicating the effectiveness of the newly applied bio-inspired optimizers.

It was observed that the R^2^statistics is not consistent with others (e.g., RMSE, MAE and NSE). Also discussed by Mahmood Agha et al.^56^, the R^2^ term serves as an indicator of the linear relationship between observed data and corresponding model predictions. Consequently, it is not always expected that R^2^ aligns perfectly with the RMSE (Root Mean Square Error). To illustrate, consider two time series: (Yi, observed = 1, 2, 3,., 10; Yi, predicted = 20, 40, 60,., 200). Despite the R^2^ between these two series being 1, indicating a perfect fit, the RMSE value remains considerably high. It is important to note that an R^2^ value of 1 does not necessarily guarantee that a model fully captures the underlying behavior of the investigated time series.

The outcomes of the LSTM-based methods clearly reveal that choice of testing data sets (M1, M2 and M3) considerably affect the accuracy of the LSTM-based methods in predicting Epan in both stations. As also discussed by Chen et al.^57^ and Kisi et al.^58^, separating the data sets into only two parts (training and testing) may mislead the results. Data driven methods highly depend on data distribution and therefore, testing the implemented methods with different data sets is more appropriate in order to decide the best model.

Another important finding is that including Tmax and Ra variables did not always improve accuracy. This finding is in agreement with earlier research conducted by scholars like Shi et al.^59^, Adnan et al.^60,61^, and Zhang et al.^62^. These studies consistently indicated that augmenting the number of inputs does not always lead to enhanced prediction accuracy; instead, it can potentially have an adverse effect on variance. Consequently, this may give rise to more intricate models with diminished prediction performance.

This behavior is also supported by the results from this study. For instance, in Station 1 under the M3 scenario, the LSTM model using Tmin and Tmax inputs achieved a lower RMSE of 0.5508 compared to 0.7584 with Tmin and Ra and 0.6438 with Tmax and Ra. This shows that including Ra does not necessarily add predictive value. We see similar patterns consistently across hybrid models, which confirms this finding.

From a hydrological viewpoint, pan evaporation mainly depends on the vapor pressure gradient and surface energy balance, both of which are strongly influenced by air temperature. Thus, Tmin and Tmax effectively represent the atmospheric demand and thermal conditions needed for evaporation. While extraterrestrial radiation (Ra) indicates the theoretical incoming solar energy at the atmosphere’s top, it fails to consider local atmospheric conditions like cloud cover, humidity, and aerosols. These factors greatly affect the actual energy available at the surface. Consequently, Ra may not offer any useful information beyond what temperature variables already provide, leading to redundancy in data-driven models.

Additionally, using Ra can be helpful in situations with limited data since it can be calculated based on geographical and astronomical relationships without direct measurements. However, since it does not capture surface-level variability, its ability to improve model performance may be limited. Therefore, the strong performance of temperature-based input combinations shows that we can make reliable pan evaporation predictions using scientifically meaningful and widely available variables, which enhances the robustness and practical use of the proposed models.

## Conclusions and recommendations

The current study offers valuable insights into predicting pan evaporation using machine learning techniques. It focuses on two new hybrid deep learning models: LSTM with Artificial Protozoa Optimizer (LSTM-APO) and LSTM with Dung Beetle Optimizer (LSTM-DBO). The evaluation of these models shows clear benefits over other methods when predicting monthly pan evaporation.

The numerical evaluation results indicate that both LSTM-APO and LSTM-DBO consistently outperformed the standard LSTM and the regular hybrid models (LSTM-GWO and LSTM-HHO) in predicting pan evaporation across both stations and all test data sets (M1, M2, and M3). The LSTM-APO achieved the most significant improvements, reducing RMSE and MAE by over 40%. It also increased R² and NSE by about 8 to 9% compared to the best-performing standard LSTM cases. The LSTM-DBO provided substantial improvements as well, achieving error reductions of 20 to 30% and greater stability in prediction. These findings highlight the accuracy and reliability of the proposed APO and DBO hybrid models.

The numerical evaluation results further emphasize that using advanced metaheuristic algorithms in deep learning models greatly improves precision and generalization. The LSTM-APO stood out as the most accurate and stable model, while the LSTM-DBO delivered competitive and consistent results. These hybrid models thus represent promising tools for practical applications in water resource management, agriculture, and environmental conservation, especially when input data is sparse or limited. These findings show how useful the proposed models are for local stakeholders, such as water resource managers, agricultural planners, and environmental agencies. This is especially true in areas where data is limited.

Developing models that can handle real-time meteorological data would be valuable for operational use. The integration of real-time data feeds into these hybrid models could facilitate timely pan evaporation predictions, supporting adaptive water resource management and decision-making processes. Furthermore, evaluating the transferability of the LSTM-APO and LSTM-DBO models to different regions with varying climatic conditions is crucial. Such investigations would help to verify the models’ robustness and generalizability and contribute to the sustainable management of water resources on a broader scale.

However, it is important to note that this study relies on limited input variables and monthly data. This may not fully capture short-term variability and complex hydrological processes. Future studies should include more meteorological variables, such as humidity and wind speed, and use data with higher temporal resolution to improve model performance and physical representation.

## Supplementary Information

Below is the link to the electronic supplementary material.

Supplementary Material 1

## Data Availability

The data used in this study are available from the corresponding author upon reasonable request.

## References

[1] Zhou, Z. et al. Hierarchically porous carbonized *Pleurotus eryngii* based solar steam generator for efficient wastewater purification. *Renew. Energy* 118987. 10.1016/J.RENENE.2023.118987 (2023).

[2] Brady, E. et al. The connected isotopic water cycle in the community earth system model version 1. *J. Adv. Model. Earth Syst.* **11**(8), 2547–2566. 10.1029/2019MS001663 (2019).

[3] Chang, F. J., Chang, L. C., Kao, H. S. & Wu, G. R. Assessing the effort of meteorological variables for evaporation estimation by self-organizing map neural network. *J Hydrol (Amst)* **384**(1–2), 118–129. 10.1016/J.JHYDROL.2010.01.016 (2010).

[4] Khalifeh Soltani, S. R. et al. Predicting effect of floating photovoltaic power plant on water loss through surface evaporation for wastewater pond using artificial intelligence: A case study. *Sustain. Energy Technol. Assess.* **50**, 101849. 10.1016/J.SETA.2021.101849 (2022).

[5] Kim, M. S., Cha, D., Lee, S. M., Jeong, H. & Lee, C. Prediction of brine evaporation rate in a pond: Development of different models under controlled meteorological conditions and comparative evaluation. *Desalination* **551**, 116415. 10.1016/J.DESAL.2023.116415 (2023).

[6] Goyal, M. K., Bharti, B., Quilty, J., Adamowski, J. & Pandey, A. Modeling of daily pan evaporation in sub tropical climates using ANN, LS-SVR, Fuzzy Logic, and ANFIS. *Expert Syst. Appl.* **41**(11), 5267–5276. 10.1016/J.ESWA.2014.02.047 (2014).

[7] Majhi, B. & Naidu, D. Pan evaporation modeling in different agroclimatic zones using functional link artificial neural network. *Inf. Process. Agric.* **8**(1), 134–147. 10.1016/J.INPA.2020.02.007 (2021).

[8] Majhi, B., Naidu, D., Mishra, A. P. & Satapathy, S. C. Improved prediction of daily pan evaporation using Deep-LSTM model. *Neural Comput. Appl.* **32** (12), 7823–7838. 10.1007/s00521-019-04127-7 (2020).

[9] Guan, Y. et al. “A novel approach for predicting daily pan evaporation in the coastal regions of Iran using support vector regression coupled with krill herd algorithm model,”. *Theor. Appl. Climatol.* **142**(1–2), 349–367. 10.1007/s00704-020-03283-4 (2020).

[10] Keshtegar, B., Heddam, S., Sebbar, A., Zhu, S. P. & Trung, N. T. SVR-RSM: A hybrid heuristic method for modeling monthly pan evaporation. *Environ. Sci. Pollut. Res.* **26**(35), 35807–35826. 10.1007/s11356-019-06596-8 (2019). 10.1007/s11356-019-06596-8 31705408

[11] Wang, H. et al. A novel nonlinear Arps decline model with salp swarm algorithm for predicting pan evaporation in the arid and semi-arid regions of China. *J Hydrol (Amst)* **582**, 124545. 10.1016/j.jhydrol.2020.124545 (2020).

[12] Apak, S., Kilinc, H. C. & Yurtsever, A. Streamflow prediction using an incremental attention network with LSTM and chaos optimization techniques. *Ain Shams Eng. J.* **16**(11), 103664 (2025).

[13] Sarıgöl, M., Katipoğlu, O. M. & Dalkilic, H. Y. Applying data-driven modeling for streamflow prediction in semi-arid watersheds: A comparative evaluation of machine learning and deep learning methodologies. *Pure Appl. Geophys.* **181**(12), 3561–3589 (2024).

[14] Woo, D. K. Estimating actual evapotranspiration from widely available meteorological data with a hybrid CNN–LSTM. *Agric. Water Manag.* **323**, 110078 (2026).

[15] Jayasinghe, W. J. M. L. P., Deo, R. C., Ghahramani, A., Ghimire, S. & Raj, N. Development and evaluation of hybrid deep learning long short-term memory network model for pan evaporation estimation trained with satellite and ground-based data. *J. Hydrol.*10.1016/j.jhydrol.2022.127534 (2022).

[16] Houénafa, S. E., Johnson, O., Ronoh, E. K. & Moore, S. E. Hybridization of stochastic hydrological models and machine learning methods for improving rainfall-runoff modelling. *Results Eng.*, 25, 104079. (2025).

[17] Zerouali, B. et al. Enhancing water security through advanced modeling:integrating deep learning and a novel metaheuristic optimization algorithm for accurate pan evaporation prediction. *UA Water Infrastruct. Ecosyst. Soc.* **74**(1), 18–35 (2025).

[18] Alsumaiei, A. A. Hybrid residual modeling of pan evaporation in hyper-arid climates: Benchmarking interpretable neural architectures against physical drivers. *J. Hydrol. Reg. Stud.* **60**, 102572 (2025).

[19] Al-Juboori, A. M. Modeling daily pan evaporation using a CCNN-GLM hybrid model. *J. Water Manage. Model.*10.14796/JWMM.C551 (2025).

[20] Farzad, R., Sharafati, A., Ahmadi, F. & Hosseini, S. A. Reservoir evaporation prediction with integrated development of deep neural network models and meta-heuristic algorithms (Case study: Dez Dam). *Earth Sci. Inf.* **18** (2), 210 (2025).

[21] Shadkani, S., Hashemi, S., Pak, A. & Lahijan, A. B. Random forest and multilayer perceptron hybrid models integrated with the genetic algorithm for predicting pan evaporation of target site using a limited set of neighboring reference station data. *Earth Sci. Inform.* **17**(2), 1261–1280 (2024).

[22] Rong, Y. et al. A novel hybrid deep learning framework for evaluating field evapotranspiration considering the impact of soil salinity. *Water Resour. Res.* **60** (9), e2023WR036809 (2024).

[23] Marouane, B., Mu’azu, M. A. & Petroselli, A. Prediction of reservoir evaporation considering water temperature and using ANFIS hybridized with metaheuristic algorithms. *Earth Sci. Inf.* **17** (2), 1779–1798 (2024).

[24] Achite, M. et al. Comparative assessment of standalone and hybrid deep neural networks for modeling daily pan evaporation in a semi-arid environment. *Sci. Rep.* **15** (1), 20179 (2025). 40542056 10.1038/s41598-025-05985-z PMC12181372

[25] Wang, X. et al. Artificial Protozoa Optimizer (APO): A novel bio-inspired metaheuristic algorithm for engineering optimization. *Knowl. Based Syst.* **295**, 111737 (2024).

[26] Xue, J. & Shen, B. Dung beetle optimizer: A new meta-heuristic algorithm for global optimization. *J. Supercomput.* **79**(7), 7305–7336 (2023).

[27] Ding, X. & Li, X. Monitoring of the water-area variations of Lake Dongting in China with ENVISAT ASAR images. *Int. J. Appl. Earth Obs. Geoinf.* **13**(6), 894–901 (2011).

[28] Guo, R., Zhu, Y. & Liu, Y. A comparison study of precipitation in the Poyang and the Dongting Lake Basins from 1960–2015. *Sci. Rep.* **10**(1), 3381 (2020). 32099049 10.1038/s41598-020-60243-8 PMC7042326

[29] Geng, M. et al. Evaluation and variation trends analysis of water quality in response to water regime changes in a typical river-connected lake (Dongting Lake), China. *Environ. Pollut.* **268**, 115761 (2021). 33035913 10.1016/j.envpol.2020.115761

[30] Yu, Y. et al. Hydromorphological processes of Dongting Lake in China between 1951 and 2014. *J. Hydrol.* **562**, 254–266 (2018).

[31] Wang, H. et al. Evaluation of ecohydrological regime and its driving forces in the Dongting Lake, China. *J. Hydrol. Reg. Stud.* **41**, 101067 (2022).

[32] Peng, Y., He, G., Wang, G. & Cao, H. Surface water changes in Dongting Lake from 1975 to 2019 based on multisource remote-sensing images. *Remote Sens.* **13** (9), 1827 (2021).

[33] Hochreiter, S. & Schmidhuber, J. Long Short-Term Memory. *Neural Comput.* **9**(8), 1735–1780. 10.1162/neco.1997.9.8.1735 (1997). 9377276 10.1162/neco.1997.9.8.1735

[34] Kilinc, H. C. & Yurtsever, A. Short-term streamflow forecasting using hybrid deep learning model based on grey wolf algorithm for hydrological time series. *Sustainability* **14** (6), 3352 (2022).

[35] Hu, Y. et al. Investigate the rainfall-runoff relationship and hydrological concepts inside LSTM. *Environmental Modelling & Software*, 192, 106527. (2025).

[36] Luo, J., Zhu, D. & Li, D. Classification-enhanced LSTM model for predicting river water levels. *J. Hydrol.* **650**, 132535 (2025).

[37] Granata, F., Di Nunno, F. & de Marinis, G. Stacked machine learning algorithms and bidirectional long short-term memory networks for multi-step ahead streamflow forecasting: A comparative study. *J. Hydrol.* **613**, 128431. 10.1016/j.jhydrol.2022.128431 (2022).

[38] Kao, I. F., Liou, J. Y., Lee, M. H. & Chang, F. J. Fusing stacked autoencoder and long short-term memory for regional multistep-ahead flood inundation forecasts. *J. Hydrol.* **598**, 126371. 10.1016/j.jhydrol.2021.126371 (2021).

[39] Yazdan, M. M. S., Saki, S. & Kumar, R. Untangling energy consumption dynamics with renewable energy using recurrent neural network. *Analytics* **2**, 132–145. 10.3390/analytics2010008 (2023).

[40] Heidari, A. A. et al. Harris hawks optimization: Algorithm and applications. *Future Gener. Comput. Syst.* **97**, 849–872. 10.1016/j.future.2019.02.028 (2019).

[41] Houssein, E. H., Mohamed, M., Younis, E. M. & Mohamed, W. M. A hybrid Harris Hawks Optimization with Support Vector Regression for air quality forecasting. *Sci. Rep.* **15** (1), 2275 (2025). 39824922 10.1038/s41598-025-86275-6 PMC11742066

[42] Zhang, Y., Liu, R., Wang, X., Chen, H. & Li, C. Boosted binary harris hawks optimizer and feature selection. *Eng Comput.* (2020).

[43] Rammurthy, D. & Mahesh, P. Whale harris hawks optimization based deep learning classifier for brain tumor detection using mri-images. *J. King Saud Univ. Comput. Inf. Sci.* (2020).

[44] Peng, L., Cai, Z., Heidari, A. A., Zhang, L. & Chen, H. Hierarchical Harris hawks optimizer for feature selection. *J. Adv. Res.*10.1016/j.jare.2023.01.014 (2023) (**ISSN 2090 – 1232**). 36690206 10.1016/j.jare.2023.01.014 PMC10658428

[45] Mirjalili, S., Mirjalili, S. M., Lewis, A. & Grey Wolf Optimizer,. Grey Wolf Optimizer. *Adv. Eng. Softw.* **69**, 46–61. 10.1016/j.advengsoft.2013.12.007 (2014).

[46] Alnowaiser, K., Saber, A., Hassan, E. & Awad, W. A. An optimized model based on adaptive convolutional neural network and grey wolf algorithm for breast cancer diagnosis. *PloS One* **19**(8), e0304868 (2024). 39159151 10.1371/journal.pone.0304868 PMC11332925

[47] Rajput, S. S. & S-Gwo-Fh S-GWO-FH: Sparsity-based grey wolf optimization algorithm for face hallucination. *Soft Comput.* **26** (18), 9323–9338. 10.1007/s00500-022-07250-1 (2022).

[48] Li, B. J. et al. Monthly runoff forecasting using variational mode decomposition coupled with Gray Wolf Optimizer-based long short-term memory neural networks. *Water Resour. Manage.* **36**, 2095–2115. 10.1007/s11269-022-03133-0 (2022).

[49] Tikhamarine, Y., Souag-Gamane, D., Ahmed, A. N., Kisi, O. & El-Shafie, A. Improving artificial intelligence models accuracy for monthly streamflow forecasting using grey wolf optimization (GWO) algorithm. *J. Hydrol.* **582**, 124435. 10.1016/j.jhydrol.2019.124435 (2020).

[50] Elamy, M. I., Abd Elaziz, M., Al-Betar, M. A., Fathy, A. & Elmahdy, M. Enhanced random vector functional link based on artificial protozoa optimizer to predict wear characteristics of Cu-ZrO2 nanocomposites. *Results Eng.* **24**, 103007 (2024).

[51] Xie, X., Gao, Y. & Zhang, Y. An improved artificial protozoa optimizer for CNN architecture optimization. *Neural Netw.* **187**, 107368 (2025). 40112636 10.1016/j.neunet.2025.107368

[52] Tang, W., Qin, H. & Kang, S. Dung beetle optimizer based on mean fitness distance balance and multi-strategy fusion for solving practical engineering problems. *Sci. Rep.* **15**(1), 26389 (2025). 40691680 10.1038/s41598-025-02937-5 PMC12279994

[53] Liu, J., Zhang, Y., Wen, K. & Ding, Y. Analysis of different neural network models based on variational mode decomposition and Dung beetle optimizer algorithm for the prediction of air-conditioning energy consumption in multifunctional complex large public buildings. *Energy Build.* **334**, 115518 (2025).

[54] Pan, J., Jing, B., Jiao, X. & Wang, S. Analysis and application of grey wolf optimizer-long short-term memory. *IEEE Access* **8**, 121460–121468. 10.1109/ACCESS.2020.3006499 (2020).

[55] Latha, P. & Kumaresan, P. Deep learning model optimization for crop prediction and recommendation using harris hawks optimization. *Environ. Res. Commun.* **7**(4), 045008 (2025).

[56] Mahmood Agha, O. M., Ahmed, K. A. & Naser, A. I. Hydrological drought characteristics prediction using traditional models for the Great Zab River in Iraq: OMAM Agha et al. *Theor. Appl. Climatol.* **156**(5), 286 (2025).

[57] Chen, J. et al. Improved data splitting methods for data-driven hydrological model development based on a large number of catchment samples. *J. Hydrol.* **613**, 128340 (2022).

[58] Kisi, O. et al. Water quality prediction of the Yamuna River in India using hybrid neuro-fuzzy models. *Water* **15**, 1095. 10.3390/w15061095 (2023).

[59] Shi, J., Guo, J. & Zheng, S. Evaluation of hybrid forecasting approaches for wind speed and power generation time series. *Renew. Sustain. Energy Rev.* **16**, 3471–3480 (2012).

[60] Adnan, R. M. et al. Comparison of LSSVR, M5RT, NF-GP, and NF-SC models for predictions of hourly wind speed and wind power based on cross-validation. *Energies* **12**, 329. 10.3390/en12020329 (2019a).

[61] Adnan, R. M. et al. Daily streamflow prediction using optimally pruned extreme learning machine. *J. Hydrol.* **577**, 123981. 10.1016/j.jhydrol.2019b.123981 (2019bb).

[62] Zhang, D., Peng, X., Pan, K. & Liu, Y. A novel wind speed forecasting based on hybrid decomposition and online sequential outlier robust extreme learning machine. *Energy Convers. Manag.* **180**, 338–357 (2019).

