# Supplementary material for: Metaheuristic-enhanced deep learning for monthly pan evaporation prediction under limited climatic data
Source: Sci Rep. 2026 May 2;16:14039. doi: 10.1038/s41598-026-51071-3 (PMC13135511; doi:10.1038/s41598-026-51071-3)
Supplement: Supplementary file 1 — Supplementary Material 1 [file 41598_2026_51071_MOESM1_ESM.docx]

**Supplementary materials**

**Table S1.** Computational workflow of the hybrid LSTM-GWO model. The GWO is used to tune the hyperparameters of the LSTM model. Each solution vector X_i_ ​ corresponds to a candidate set of LSTM hyperparameters, and the optimal solution X^∗^ represents the best-performing configuration obtained by minimizing the objective function (RMSE).

| **Algorithm 1. LSTM-GWO Pseudocode** |
| --- |
| Input:  Meteorological variables X, pan evaporation Y  Hyperparameter space H  Population size N, maximum iterations T  Output:  Optimized LSTM model and predictions  Begin  Initialize wolf population Xi (i = 1 to N) from H  For each Xi do  Train LSTM model using Xi  Compute fitness = RMSE(Y_pred, Y_true)  End For  Identify α (best), β, δ solutions  For t = 1 to T do  For each Xi do  Update Xi using GWO position equations  If Xi is outside bounds then  Adjust Xi within bounds  End If  End For  Evaluate fitness of all Xi  Update α, β, δ  End For  Set X* = α  Train final LSTM using X*  Predict pan evaporation for test data  Return optimized model and predictions  End |

**Table S2.** Computational workflow of the hybrid LSTM-HHO model. The HHO is used to tune the hyperparameters of the LSTM model. Each solution vector X_i_ ​ corresponds to a candidate set of LSTM hyperparameters, and the optimal solution X^∗^ represents the best-performing configuration obtained by minimizing the objective function (RMSE).

| **Algorithm 2. LSTM-HHO Pseudocode** |
| --- |
| Input:  Meteorological variables X, pan evaporation Y  Hyperparameter space H  Population size N, maximum iterations T  Output:  Optimized LSTM model and predictions  Begin  Initialize hawk population Xi (i = 1 to N)  For each Xi do  Train LSTM model using Xi  Compute fitness = RMSE(Y_pred, Y_true)  End For  Identify best solution Xrabbit  For t = 1 to T do  For each Xi do  Compute escaping energy E  If \|E\| ≥ 1 then  Perform exploration phase  Else  Perform exploitation phase  End If  If Xi is outside bounds then  Adjust Xi within bounds  End If  End For  Evaluate fitness  Update Xrabbit  End For  Set X* = Xrabbit  Train final LSTM using X*  Predict pan evaporation  Return optimized model and predictions  End |

**Table S3.** Computational workflow of the hybrid LSTM-APO model. The APO is used to tune the hyperparameters of the LSTM model. Each solution vector X_i_ ​ corresponds to a candidate set of LSTM hyperparameters, and the optimal solution X^∗^ represents the best-performing configuration obtained by minimizing the objective function (RMSE).

| **Algorithm 3. LSTM-APO Pseudocode** |
| --- |
| Input:  Meteorological variables X, pan evaporation Y  Hyperparameter space H  Population size N, maximum iterations T  Output:  Optimized LSTM model and predictions  Begin  Initialize protozoa population Xi (i = 1 to N)  For each Xi do  Train LSTM model using Xi  Compute fitness = RMSE(Y_pred, Y_true)  End For  Identify best solution Xbest  For t = 1 to T do  For each Xi do  Update Xi using APO mechanisms:  Autotrophic and heterotrophic behaviors  If Xi is outside bounds then  Adjust Xi within bounds  End If  End For  Evaluate fitness  Update Xbest  End For  Set X* = Xbest  Train final LSTM model  Predict pan evaporation  Return optimized model and predictions  End |

**Table S4.** Computational workflow of the hybrid LSTM-DBO model. The DBO is used to tune the hyperparameters of the LSTM model. Each solution vector X_i_ ​ corresponds to a candidate set of LSTM hyperparameters, and the optimal solution X^∗^ represents the best-performing configuration obtained by minimizing the objective function (RMSE).

| **Algorithm 4. LSTM-DBO Pseudocode** |
| --- |
| Input:  Meteorological variables X, pan evaporation Y  Hyperparameter space H  Population size N, maximum iterations T  Output:  Optimized LSTM model and predictions  Begin  Initialize dung beetle population Xi (i = 1 to N)  For each Xi do  Train LSTM model using Xi  Compute fitness = RMSE(Y_pred, Y_true)  End For  Identify best solution Xbest  For t = 1 to T do  For each Xi do  Update Xi using DBO strategies:  Rolling, foraging, reproduction  If Xi is outside bounds then  Adjust Xi within bounds  End If  End For  Evaluate fitness  Update Xbest  End For  Set X* = Xbest  Train final LSTM model  Predict pan evaporation  Return optimized model and predictions  End |
